# 6-*O*-alkyl 4-methylumbelliferyl-β-D-glucosides as selective substrates for GBA1 in the discovery of glycosylated sterols

**DOI:** 10.1016/j.jlr.2024.100670

**Published:** 2024-10-10

**Authors:** Stef Bannink, Kateryna O. Bila, Joosje van Weperen, Nina A.M. Ligthart, Maria J. Ferraz, Rolf G. Boot, Daan van der Vliet, Daphne.E.C. Boer, Herman S. Overkleeft, Marta Artola, Johannes M.F.G. Aerts

**Affiliations:** 1 Medical Biochemistry, Leiden Institute of Chemistry (LIC), Leiden University, RA Leiden, The Netherlands; 2 Molecular Physiology, Leiden Institute of Chemistry (LIC), Leiden University, RA Leiden, The Netherlands; 3 Bio-organic Synthesis, Leiden Institute of Chemistry (LIC), Leiden University, RA Leiden, The Netherlands

**Keywords:** JLR, cholesterol, phytosterols, cerebrosides, glycolipids, enzymology, Gaucher disease, lysosomal storage disease, glycosidase, enzymatic assay, 4-methylumbelliferyl substrate

## Abstract

Gaucher disease (GD) is a lysosomal storage disorder (LSD) resulting from inherited glucocerebrosidase (GBA1) deficiency. GD diagnosis relies on GBA1 activity assays, typically employing 4-methylumbelliferyl-β-D-glucopyranoside (4MU-β-Glc) as fluorogenic substrate. However, these assays suffer from background 4MU release by the non-lysosomal GBA2 and cytosolic GBA3 enzymes. Here we developed GBA1-selective fluorogenic substrates by synthesizing a series of 6-*O*-acyl-4MU-β-Glc substrates with diverse fatty acid tails. Because of the chemical and enzymatic instability of the ester bonds, analogs of 6-*O*-palmitoyl-4MU-β-Glc (3) with different chemical linkages were synthesized. 6-*O-*alkyl-4MU-β-Glc 9, featuring an ether linkage, emerged as the most optimal GBA1 substrate, exhibiting both a low *K*_m_ and compared to substrate 3 a high V_max_. Importantly, substrate 9 is not hydrolyzed by GBA2 and GBA3 and therefore acts as a superior substrate for GD diagnosis. Plants contain glycosyl phytosterols (campesterol, β-sitosterol, and sigmasterol) that may also be acylated at C-6. LC-MS/MS analysis revealed that 6-*O*-acylated and regular glycosylcholesterol (HexChol) tend to be increased in spleens of patients with GD. Moreover, significant increases in 6-*O*-acyl-glycosyl-phytosterols were detected in GD spleens. Our findings suggest uptake of (6-*O*-acyl)-glycosyl-phytosterols from plant food and subsequent lysosomal processing by GBA1, and comprise the first example of accumulation of an exogenous class of glycolipids in GD. Excessive exposure of rodents to glycosylated phytosterols has been reported to induce manifestations of Parkinson’s disease (PD). Further investigation is warranted to determine whether (6-*O*-acyl)-glycosyl-phytosterols could contribute to the enigmatic link between inherited defects in GBA1 and the risk for PD.

The lysosomal retaining β-D-glucosidase (EC. 3.2.1.45), glucocerebrosidase (*aka* GCase or GBA1), is a 497-amino acid glycoprotein responsible for removing the glucose moiety from glucosylceramide (GlcCer), an essential step in lysosomal breakdown of most glycosphingolipids (GSLs) (1, 2) Mutations in the *GBA1* gene at locus 1q21 can lead to cellular deficiency of glucocerebrosidase activity and cause Gaucher disease (GD). In patients with GD, there is a prominent lysosomal accumulation of GlcCer in tissue macrophages, which transform into enlarged viable “Gaucher cells”, a process accompanied by the manifestation of a wide range of symptoms (3, 4, 5). The most frequent phenotypic manifestation of GD in Caucasian populations is the non-neuronopathic variant of the disease, often indicated as type 1 (5). More severe neuronopathic variants of GD, such as type 2 and 3, also occur. The heteroallelic presence of the N370S GBA1 substitution is associated with type 1 GD, while the homoallelic presence the L444P GBA1 substitution is associated with the pathology of the central nervous system (2, 6). Currently, there are two registered therapies for the treatment of type 1 GD: enzyme replacement therapy (ERT) and substrate reduction therapy (SRT) (7, 8). ERT involves the intravenous administration of recombinant rhGBA1 every two weeks, resulting in a decrease in visceral symptoms including organomegaly and hematological abnormalities. Due to the inability of the enzyme to pass the blood–brain barrier, ERT does not correct neurological symptoms present in neuropathic variants of GD. SRT, concerning the daily oral administration of glucosylceramide synthase (GCS) inhibitors (Miglustat or Eliglustat) focuses on reducing GSL synthesis. The residual GBA1 activity of Patients with GD is then sufficient and prevents accumulation of GlcCer, as a consequence of GCS inhibition (7, 8). It has been recognized that mutations in the *GBA1* gene, even when only one allele has been mutated, pose a risk factor for developing Parkinson’s disease (PD) (9, 10). However, the link between GBA1 defects and PD is still enigmatic.

Ideally, early therapeutic intervention in GD is crucial to prevent severe complications like massive fibrous splenomegaly, avascular necrosis, and pathological fractures (4). Recently an international network of expert centers has reviewed type 1 GD laboratory diagnosis and recommends the measurement of reduced GBA1 activity as the central confirmatory assessment of the disease (5). Genotyping and the analysis of circulating biomarkers are suggested as secondary tests. The most common method for laboratory diagnosis of GD involves measuring GBA1 activity in white blood cells or fibroblasts using the artificial fluorogenic substrate, 4-methylumbelliferyl-β-D-glucopyranoside (4MU-β-Glc), and benchmarking it against a standard (5). However, the presence of both the non-lysosomal cytosol-facing membrane-bound GBA2 and broad substrate-specific cytosolic GBA3 enzymes in materials creates a background of released 4MU, thereby complicating the analysis (11, 12). The use of specific inhibitors of GBA1 could provide clearer insight into the contribution of GBA1 to the released 4MU from 4MU-β-Glc. The restricted availability of highly specific GBA1 inhibitors, e.g. ME656 (13), limits the use of the more sophisticated GBA1 activity measurement in biological materials. Therefore, there is a need for a fluorogenic 4MU-substrate specific to GBA1. It has been previously established that 4-methylumbelliferyl-β-D-xylopyranoside (4MU-β-Xyl) is a specific substrate of GBA1; however, it exhibits a significantly lower V_max_ (∼50 fold) when compared to 4MU-β-Glc (14). In terms of specificity, the CH_2_OH group at C-6 of the sugar appears highly relevant. This observation is further supported by C-6-modified cyclophellitol-based inhibitors (carbohydrate numbering) which selectively target GBA1 over GBA2 and GBA3 (15, 16). The same approach of extending the C-6 position of the sugar has also been used by Vocadlo and coworkers in the design and synthesis of his fluorescence-quenched substrates for live cell imaging of human glucocerebrosidase activity (17). Building upon this knowledge, we embarked on the design and synthesis of a series of 6-*O-*acylated and alkylated 4MU-β-Glc derivatives, intending to develop candidate substrates that specifically interact with GBA1. The outcome of our investigation is here reported and was instrumental in the discovery of natural 6-*O*-acyl-glycosyl-sterols and 6-*O*-acyl-glycosyl-phytosterols as natural substrates of GBA1. Of interest, the occurrence of glucosylated sterols in man was earlier described by Akiyama and colleagues (18) and Marques and coworkers (19) as well as that of plant-type glucosylated β-sitosterol (20).

## Materials and methods

Cerezyme® (rhGBA, 1.363 mg/ml) was a kind gift from Sanofi Genzyme. 4-Methylumbelliferyl-β-D-glucopyranoside (4MU-β-Glc) was purchased from Glycosynth (Winwick Quay). 4MU substrates 2–10 and internal heavy isotope standard 31 were synthesized as described in the supporting information. Triton X-100 was purchased from Sigma-Aldrich. Taurocholic acid sodium salt and dimethyl sulfoxide (DMSO) were purchased from EMD Millipore Corporation (Billerica).

Spleens from Patients with GD and (non-GD) controls had been obtained with consent and were used in prior research (21, 22).

### Fluorogenic assays for 4MU substrates 1–5 and 6-*O*-acylated and -alkylated substrates 6-10

The in vitro enzyme activity of rhGBA1 (Cerezyme®) using substrates 1–10 was determined by measuring the release of the fluorescent 4-methylumbelliferyl (23). Substrate mixes were made in 150 mM McIlvaine buffer pH 5.2 containing 0.2% (w/v) sodium taurocholate. Cerezyme® was diluted 1:400 in 25 mM KPI pH 5.2% and 0.1% (v/v) Triton X-100. Per well 12.5 μl enzyme mix, 12.5 μl 150 mM McIlvaine buffer, and 100 μl substrate mix were incubated for 30 min at 37°C. The reaction was stopped by adding 200 μl of the stop buffer 1 M glycine-NaOH pH 10.3. As a standard and for quantification of the obtained signals 1 nmol 4MU was added. GraphPad Prism 9 was used to analyze the results. Fluorescence intensities in the fluorogenic substrate assays were measured with a fluorimeter LS55 (PerkinElmer, Beaconsfield, UK) at λ_ex_ 366 nm and λ_em_ 445 nm plus slit_ex_ 10 nm and slit_em_ 3.0 nm. For 4MU substrates, 2–5 substrate mixtures with 0.25, 0.5, 1.0, 1.5, 2.0, 2.5, 3.0, 3.5 and 4.0 mM substrate were prepared as described above with additional 0.1% (v/v) Triton X-100 and a maximum of 0.8% (v/v) DMSO. For 4MU substrates 6–10 substrate mixtures with 0.2, 0.4, 0.6, 0.8, 1.0, 1.5 2.0, 3.0, and 4.0 mM substrate were prepared with additional 0.5% (v/v) Triton X-100 and a maximum of 10% (v/v) DMSO.

### Determination of pH-optima for 4MU-β-Glc 1, 3 and 9

For the pH curves, substrate mixes of 1, 3, and 9 in 150 mM McIlvaine buffer with the appropriate pH (pH 3.0, 4.0, 4.6, 4.8, 5.0, 5.2, 5.4, 5.6, 6.0, 7.0 or 8.0) were prepared using the fluorogenic substrate assay conditions described above except for a substrate and DMSO concentration of 1 mM and 2.5% (v/v), respectively.

### Cell culture

HEK293T cells overexpressing GBA2 (GBA/GBA2 KO) and HEK293T cells overexpressing GBA3 (GBA/GBA2 KO) were cultured in DMEM medium (Sigma-Aldrich) supplemented with 10% (v/v) FCS, 0.1% (w/v) penicillin/streptomycin and 1% (v/v) Glutamax at 37°C and 5% CO_2_. Lysates of the HEK293T cell pellets were generated by diluting the samples with 800 μl 25 mM KPI lysis buffer pH 5.8 for GBA2 or 100 mM HEPES buffer pH 7.0 for GBA3. Afterward, the lysates were sonicated 5 times for 1 s at 20% amplitude with Vibra-CellTM VCX130 (Sonics) in an ice bath.

Protein concentrations were determined with a BCA protein assay (BCA kit, Pierce, Thermo Fisher), using the Emax Plus Microplate Reader (Molecular Devices).

### Activity of substrate 9 against GBA2 and GBA3

The enzymatic activities of GBA2 and GBA3 were measured in lysates of cells overexpressing the corresponding enzyme using the same conditions as above. The 4MU substrates were tested on HEK293T cells overexpressing GBA2 dissolved in 150 mM McIlvaine buffer pH 5.8, whereas HEK293T cells overexpressing GBA3 were dissolved in 100 mM HEPES buffer pH 7.0. The fluorogenic assay conditions described above were used except for a substrate and DMSO concentration of 1 mM and 2.5% (v/v), respectively.

### Spleen lysates

Spleens from Patients with GD and (non-GD) controls were lysed using 1.0 mm glass beads with an MP biomedicals FastPrep-24 in KPI pH 6.5 supplemented with 0.1% Triton X-100: 1 g tissue per 2 ml buffer. Protein concentrations were determined with the BCA protein assay. Lysates were diluted to 30 mg protein/ml, aliquoted, and frozen at −80°C before use.

### Analysis of AcylHexSterols and HexSterols in spleen homogenates by LC-MS/MS

Prior to extraction, 5 pmol of ^13^C_6_-GlcChol and 6-*O*-palmitoyl-^13^C_6_-GlcChol (31) (used as internal standards) were added to 25 μl of homogenate. Next, lipids were extracted according to the method of Bligh and Dyer by the addition of methanol, chloroform, and water (1:1:0.9, v/v/v), and the lower phase was taken to dryness under a stream of nitrogen. Isolated lipids were further extracted by water/butanol extraction (1:1, v/v) (19). Lipids were separated using a BEH C18 reversed-phase column (2.1 × 50 mm, particle size 1.7 μm Waters Corporation) by applying an isocratic elution of mobile phases, 2-propanol: water 90:10 (v/v) containing 10 mM ammonium formate (eluent A) and methanol containing 10 mM ammonium formate (eluent B). HexSterols were eluted as previously described (19) at a flow rate of 0.25 ml/min with 10% A and 90% B for 5.5 min. The column temperature was kept at 23°C and the autosampler at 10°C. For AcylHexSterols separation, the UPLC program had a duration of 6.5 min and consisted of 30% A and 70% B at a flow rate of 0.5 ml/min. In these measurements, the column temperature and the temperature of the autosampler were kept at 40°C and 10°C, respectively, during the run. For quantitative analysis of (Acyl)-HexSterols in samples of the spleen, a method using the multiple reaction monitoring (MRM) modes was developed using the transitions described in the supplemental (supplemental Table S1). A signal-to-noise ratio of three was set for the limit of detection and the limit of quantitation was processed with a signal-to-noise ratio above 10. Calculation of the signal-to-noise ratio was done using the peak-to-peak method. LC-MS/MS measurements were performed using a Waters UPLC-Xevo-QS micro instrument (Waters, Corporation) in positive mode using an electrospray ionization source as described before (19). Data were analyzed with Masslynx 4.2 software (Waters Corporation).

## Results

**Fig. 1:**
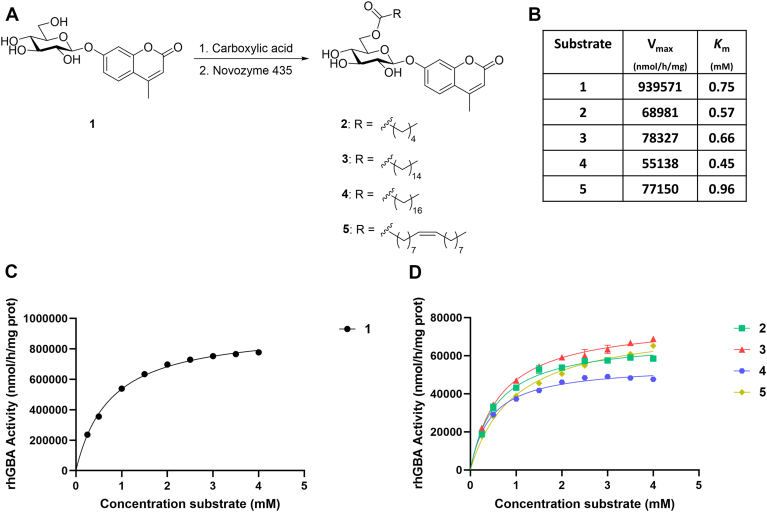
A: Enzymatic synthesis of 6-*O*-acyl-4MU-β-Glc derivatives with variable lipids (2: caproyl; 3: palmitoyl; 4: stearoyl and 5: oleoyl). B: Maximum rate of hydrolysis (V_max_) and the Michaelis constant (*K*_m_) for fluorogenic substrates 1–5. C: rhGBA activity using 4MU-β-Glc (1). D: rhGBA activity using 6-*O*-acyl-4MU-β-Glc substrates 2–5.

We first synthesized and assessed as GBA1 substrates several different 6-*O*-acyl-4MU-β-Glc derivatives (2: caproyl; 3: palmitoyl; 4: stearoyl; and 5: oleoyl, Fig. 1A). For this purpose, commercial 4MU-β-Glc was acylated by means of an enzymatic reaction catalyzed by lipase Novozyme 435.

These compounds were tested as substrates for GBA1 using pure recombinant GBA1 (rhGBA, Cerezyme®). The examination revealed that rhGBA1 retains hydrolysis activity towards substrates with extensions at C-6 (Fig. 1B, D). rhGBA1 processes 6-*O*-acyl glucosides 2–5 with about equal efficiency as it does 4MU-β-Glc, irrespective of the chain length of the 6-*O*-acyl group. When comparing the maximum hydrolysis rate (V_max_) of GBA1 with 4MU-β-Glc and with the 6-*O*-acyl glucosides 2–5, there was approximately a 12-fold decrease.

**Fig. 2:**
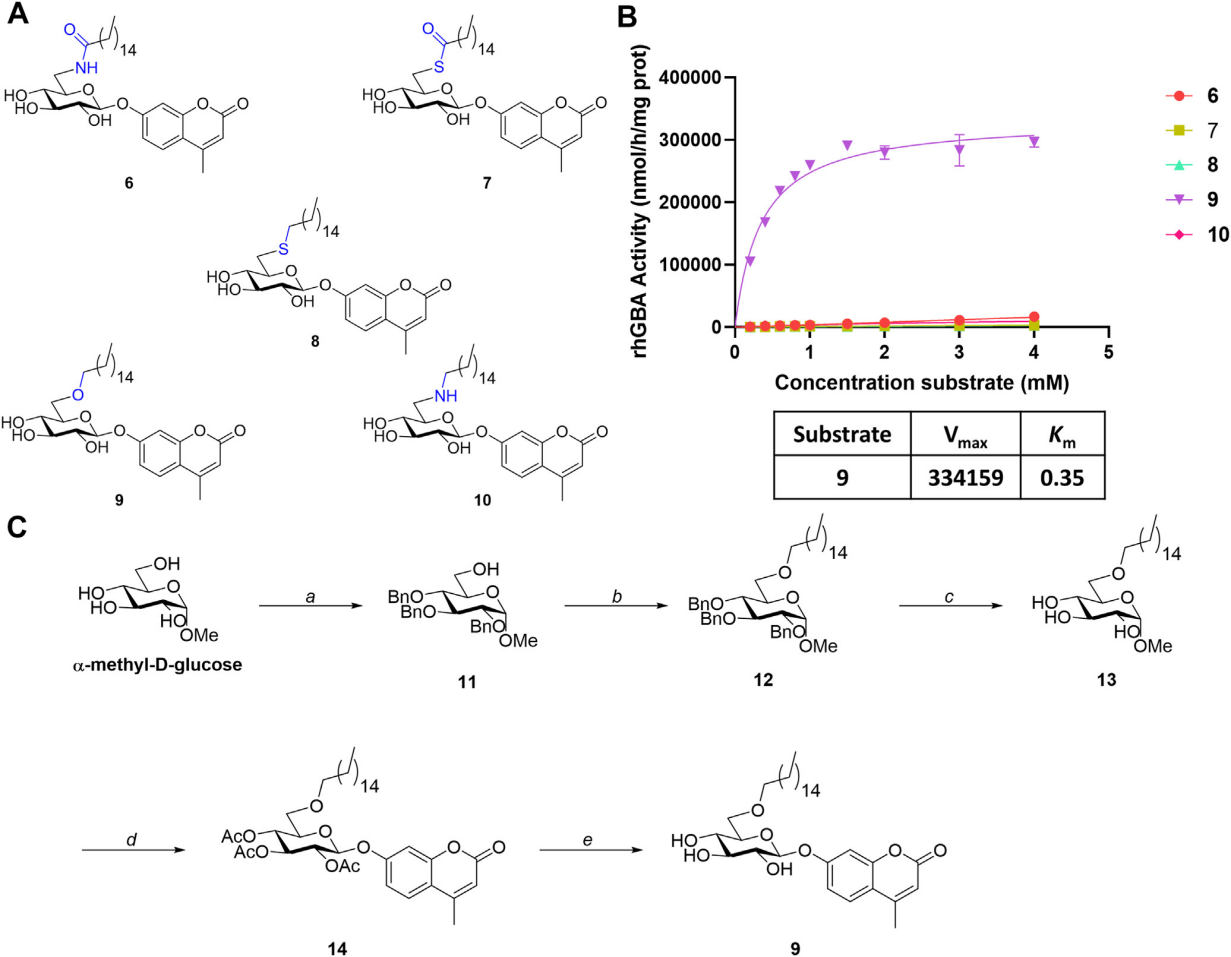
A: Chemical structures of second-generation GBA1 substrates 6–10. B: Maximum rate of hydrolysis (V_max_) and the Michaelis constant (*K*_m_) for fluorogenic substrates 6–10. C: Synthesis of 6-*O*-alkyl-4MU-β-Glc 9. Reagent and conditions: a: i. TBSCl, pyridine, 0°C – r.t., 2 h; ii. BnBr, NaH, DMF, 70°C, 3 h; iii. HCOOH/H_2_O (4:1), THF, 0°C – r.t., 3 h, 62% (3 steps). b: 1-bromohexadecane, NaH, DMF, 0°C to r.t., 16 h, 89%. c) Pd/C, H_2_, HCl, EtOAc/EtOH (1:1), r.t., 4 h, 96%. d: i. H_2_SO_4_, Ac_2_O/AcOH, 0°C – r.t., 18 h; ii. TMSBr, BiBr_3_, CH_2_Cl_2_, 0°C – r.t., 18 h; iii. 4MU, NaOH, Acetone/H_2_O (1:1), r.t., dark, 18 h, 36% (3 steps). e: NaOMe, MeOH/CH_2_Cle, r.t., 4 h, 74%.

The ester linkage in the novel compounds is intrinsically vulnerable to esterase-mediated processing in cells and cell extracts, which may be a caveat for use of the substrates in such complex biological samples. Physiologically more stable analogues of 6-*O*-acyl glucosides will not suffer from esterase-mediated processing given that these analogues are still suitable GBA1 substrates. With this in mind, a second generation of 6-*O*-acyl glucosides was synthesized (Fig. 2A) based on 6-*O*-palmitoyl-4MU-β-Glc (3). This substrate was used as a basis since it showed the most favorable kinetics independent of the chain length. Five different chemical linkages were selected: 6 with an amide linkage, 7 with a thioester linkage, 8 with a thioether linkage, 9 with an ether linkage, and 10 with an amine linkage. The synthesis of compound 9 is highlighted below while the synthesis of compounds 6–8 and 10 is described in the supplementary information.

The synthesis of compound 9 started from ɑ-methyl-D-glucose and partially protected intermediate 11 was obtained following described procedures (24). Subsequent alkylation of 11 with bromohexadecane using sodium hydride in DMF led to the formation of compound 12 in 89%. The benzyl-protecting groups were removed using standard palladium on carbon hydrogenation conditions in 96% yield. Intermediate 13 was per-acetylated using sulfuric acid in an acetic acid and acetic anhydride mixture, and the crude intermediate was subsequently brominated using TMSBr and a catalytic amount of bismuth tribromide. After workup, the unstable brominated intermediate was immediately coupled to 4-Methylumbelliferone (4MU) using NaOH to generate intermediate 14 in 37% over 3 steps. Methanolysis of the acetates using sodium methoxide gave compound 9 in 11% yield over 9 steps.

The newly synthesized 4MU-β-Glc derivatives 6–10 were subsequently assessed as substrates for rhGBA1 (Fig. 2B). Fluorogenic assays of these substrates and control samples were supplemented with 2.5% DMSO (v/v) and 0.5% Triton X-100 (v/v) to ensure proper solubility.

Surprisingly, only the ether-linked derivative 9 was processed by rhGBA1 while all other derivatives proved to be poor substrates for GBA1. The maximum rate of the enzymatic cleavage (V_max_) for 6-*O*-alkyl-4MU-β-Glc 9 was around 3-fold lower than that for 4MU-β-Glc but approximately 5-fold higher than its parent 6-*O*-palmitoyl-4MU-β-Glc substrate 3. Furthermore, the affinity of 9 for the enzyme, as indicated by the lower *K*_m_ value, is higher for 9 when compared to both 3 and 4MU-β-Glc. This indicates that the removal of the carbonyl moiety in the ester-linked substrate 3 enhances binding to the active site.

**Fig. 3:**
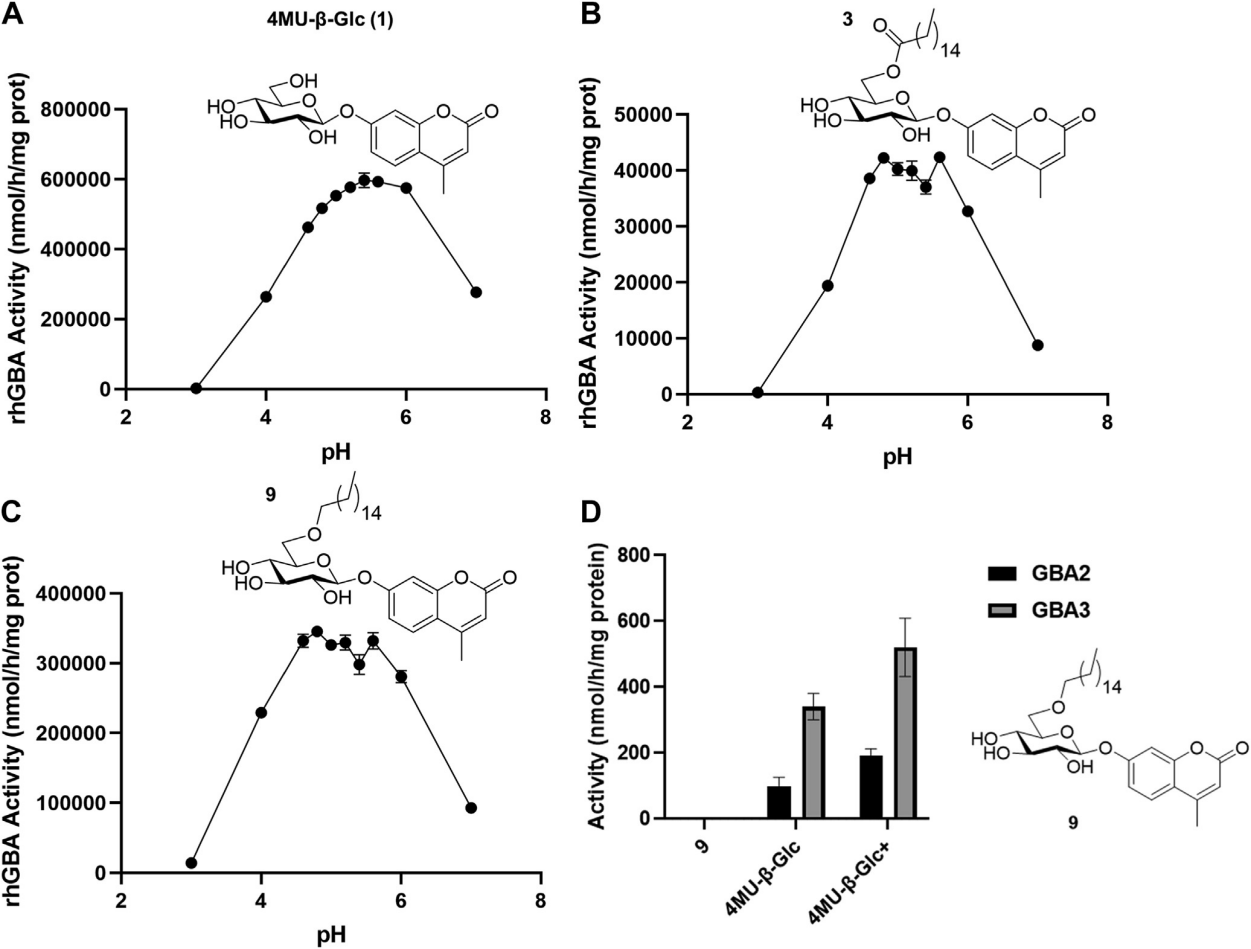
pH-dependent processing by rhGBA1 of 4MU-β-Glc 1 (A), 3 (B), and 9 (C) at 1 mM of substrate. D: Hydrolysis of 1 mM of 9 (2.5% DMSO supplemented with 0.5% Triton X-100), 1 mM of 4MU-β-Glc, and 1 mM of 4MU-β-Glc + (2.5% DMSO supplemented with 0.5% Triton X-100) by GBA2 and GBA3 incubated for 30 min at 37°C. For GBA2 activities, HEK293T cells overexpressing GBA2 with a concentration of 4.9 mg/ml and a pH of 5.8 were used, whereas for GBA3 HEK293T cells overexpressing GBA3 with a concentration of 5.4 mg/ml and an optimal pH of 7.0 were used.

The pH-optima of fluoregenic substrates 1, 3, and 9 where processing by rhGBA1 is the fastest were determined (Fig. 3A–C). While 4MU-β-Glc showed a pH-optimum of 5.4 (Fig. 3A), a broader pH-optimum between 4.8 and 5.6 was observed for substrates 3 and 9 (Fig. 3B, C). The pH-optima coincides with the acidic pH of lysosomes where GBA1 resides.

Having identified a fluorogenic substrate which reports optimally at a pH similar to those found in lysosomes under physiological conditions, the specificity for the lysosomal GBA1 was then examined using different HEK293T cells overexpressing GBA2 or GBA3 (Fig. 3D). Substrate concentrations of 1 mM were used exceeding in all cases the *K*_m_ values by two-fold. The ether-linked substrate 9 was not hydrolyzed by GBA2 and GBA3, while 4MU-β-Glc was hydrolyzed by GBA2 and GBA3 both with and without the required additives to solubilize 9, indicating that the synthesized substrate is a specific substrate for GBA1.

### Diagnosis of Gaucher disease with different 4MU substrates: GBA1 selectivity of substrate 9

The human spleen is known to be rich in the β-glucosidases GBA1, GBA2, and GBA3 (22). To see how substrate 9 behaves in a complex system we next measured enzymatic activity in lysates of spleens from non-GD control individuals and patients with GD using 4MU-β-Glc 1, the 6-*O*-alkyl-4MU-β-Glc substrate 9 and 4MU-β-Xyl as substrates. Assays were performed following pre-treatment with and without the GBA1-specific suicide inhibitor ME656 (13). The activity measured with 4MU-β-Xyl, even without ME656 treatment, was extremely low: <2 nmol/h/mg protein for control spleen lysates (supplemental Fig. S1). The activity measured with the new substrate 9 was considerably higher: 5–19 nmol/h/mg protein (Table 1). The table displays the total β-glucosidase activity in the spleen lysates toward 4MU-β-Glc 1 and 9. Importantly, when using 9 as substrate, a clearly reduced activity was observed in the GD spleen lysates compared to the control ones. Pretreatment of the spleen lysates with ME656 eliminated virtually all activity towards substrate 9. This result suggests that substrate 9 indeed allows specific assessment of GBA1 activity. Of note, the activity towards 4MU-β-Glc 1 detected in spleen lysates that were pretreated with ME656 reflects the enzymes GBA2 and GBA3 activities. This combined GBA2 and GBA3 activities in Gaucher spleen lysates is quite comparable to that in control spleen lysates.

**Table 1:** β-Glucosidase activity in non-GD spleen lysates (samples 1–5) and GD spleen lysates (samples 6–13) using 4MU-β-Glc 1 and 6-*O*-alkyl-4MU-β-Glc substrate 9, with or without pretreatment with selective and covalent GBA1 inhibitor ME656

Spleen Sample	Protein (mg/ml)	Activity substrate 1 – ME656 (nmol/h/mg protein)	Activity substrate 1 + ME656 (nmol/h/mg protein)	Activity substrate 9 – ME656 (nmol/h/mg protein)	Activity substrate 9 + ME656 (nmol/h/mg protein)
Control					
C1	65.2	22.0 ± 2.4	5.4 ± 0.4	12.2 ± 1.1	0.0 ± 0.0
C2	53.2	12.0 ± 1.6	3.8 ± 0.7	5.1 ± 0.6	−0.1 ± 0.0
C3	37.0	13.2 ± 1.8	2.4 ± 0.4	6.2 ± 1.6	0.0 ± 0.0
C4	50.0	26.5 ± 1.8	3.8 ± 0.2	19.0 ± 0.7	0.0 ± 0.0
C5	39.6	22.7 ± 0.7	12.8 ± 1.8	8.9 ± 0.2	0.2 ± 0.0
Gaucher disease					
GD1	52.6	5.0 ± 0.7	3.8 ± 0.6	0.9 ± 0.0	0.0 ± 0.0
GD2	66.5	5.0 ± 0.7	2.9 ± 0.6	1.4 ± 0.1	0.0 ± 0.0
GD3	51.4	3.2 ± 0.3	2.3 ± 0.3	0.8 ± 0.1	0.0 ± 0.0
GD4	54.7	4.7 ± 0.7	4.4 ± 0.6	0.1 ± 0.1	−0.1 ± 0.0
GD5	53.9	5.2 ± 0.5	3.9 ± 0.6	0.3 ± 0.0	−0.1 ± 0.0
GD6	52.9	2.8 ± 0.6	1.9 ± 0.6	0.4 ± 0.6	−0.1 ± 0.6
GD7	51.4	3.4 ± 0.3	2.0 ± 0.1	0.1 ± 0.0	−0.1 ± 0.0
GD8	54.6	3.2 ± 0.4	2.3 ± 0.3	0.1 ± 0.0	−0.1 ± 0.0

### Natural occurrence of 6-*O*-acyl-glucosyl-sterols and increased levels in GD spleens

**Fig. 4:**
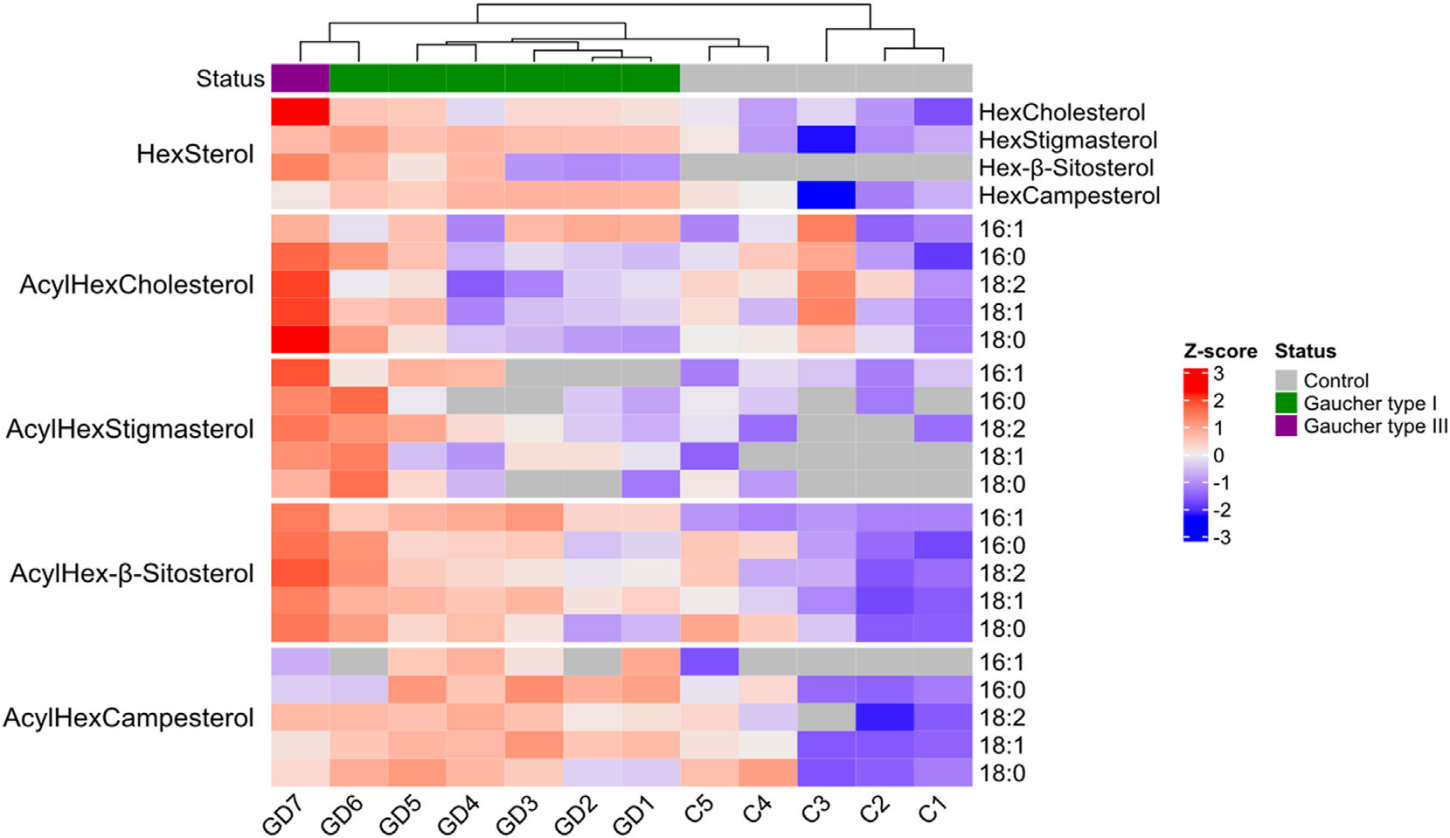
Heat map of glycosylcholesterol (HexChol) and glycosyl-stigmasterol, glycosyl-β-sitosterol, glycosyl-campesterol levels and their respective 6-*O*-acyl forms (16:0, 16:1, 18:0, 18:1 and 18:2) in spleens from non-GD and Patients with GD determined by LC-MS/MS analysis. Gaucher type 1, non-neuropathic variant of GD; Gaucher type 3, sub-acute neuronopathic GD case.

Plants contain specific sterols that can be conjugated with glucose and 6-*O*-acyl glucose, collectively named 6-*O*-acyl-sterolins (25, 26). Except for the occurrence and metabolism of glucosylcholesterol (GlcChol) and galactosylcholesterol (GalChol), grouped as glycosylcholesterol (HexChol), the existence of glycosylated sterols in humans is still poorly studied (27, 28, 29). Knowing that GBA1 is able to process compounds 3 and 9 we considered the natural occurrence of 6-*O*-acyl-glucosyl-lipids. Such 6-*O*-acyl-glucosyl-lipids might be GBA1 substrates. Plants contain high levels of glucosylated phytosterols and 6-*O*-acyl derivatives. We hypothesized that the uptake of these lipids through nutrition might lead to their accumulation in the tissues of patients with GD. We thus assessed the presence of HexChol and plant-derived phytosterols (stigmasterol, β-sitosterol, and campesterol) in GD and non-GD spleens using LC-MS/MS. As illustrated in Fig. 4, the levels of HexChol tended to be higher in GD spleens. Strikingly, also the levels of glycosyl-stigmasterol, glycosyl-β-sitosterol, and glycosyl-campesterol were found to be significantly elevated in the patients’ spleens. Most striking was the elevation of plant-derived glycosyl-β-sitosterol.

Next, we examined the presence of 6-*O*-acyl-glycosyl-cholesterol and plant 6-*O*-acyl-glycosyl-phytosterols in spleens from control subjects and Patients with GD (Fig. 4). For these measurements an appropriate structurally related standard (6-*O*-palmitoyl-^13^C_6_-GlcChol (31)) was synthesized and used. Importantly, 6-*O*-acyl-glycosyl-sterols were elevated in spleens from Patients with GD . Various 6-*O*-acyl-glycosyl-β-sitosterols were found to be increased in GD spleens as compared to control tissue (Fig. 4 and supplemental Fig. S4). The levels of various 6-*O*-acyl-glycosyl-campesterols particularly stigmasterol were lower in the examined spleens. For some 6-*O*-acyl-glycosyl-campesterols also significantly increased levels in the GD spleen were observed. As illustrated in the supplemental Fig. S3, although a similar trend was observed, analysis of 6-*O*-acyl-glycosyl-cholesterols using non-acyl ^13^C_6_-GlcChol as an internal standard did not always reach statistical significance. A similar issue was detected when analyzing 6-*O*-acyl-glycosyl-sterols (supplemental Fig. S4 versus supplemental Fig. S5). Therefore, the use of structural analogs as internal standards is recommended for the best quantification (Heat map illustrated in supplemental Fig. S6). Of note, deacylation of 6-*O*-acyl-glycosyl-sterols is feasible. As shown in supplemental Fig. S7 microwave-assisted deacylation or incubation for 10 h at alkaline conditions led to the removal of the acyl moieties.

Finally, we examined the ability of rhGBA1 to fragment glucosylated phytosterols. As shown in supplemental Fig. S8A, incubation of GlcChol, glucosyl stigmasterol and glucosyl sitosterol with rhGBA1 in the presence of the detergents Triton X-100 and taurocholate at pH 5.2 led to a comparable deglucosylation. Degradation of 6-*O*-palmitoyl-glucosyl-cholesterol or 6-*O*-palmitoyl-glucosyl-sitosterol by rhGBA1 was not prominent. Conceivably, the poor solubility of 6-*O*-acyl-glucosyl-sterols may have contributed to this.

## Discussion

We previously observed that, in contrast to the GBA2 enzyme, GBA1 tolerates a hydrophobic extension at C-6 (carbohydrate numbering) of cyclophellitol (and at C-5 according to the cyclophellitol numbering). This observation led to the design of a specific suicide inhibitor for GBA1 equipped with hydrophobic moiety at the cyclophellitol C-5 (ME656) (13). The compound ME656, binding in a reaction mechanism-based manner to the catalytic nucleophile E340 of GBA1, does not inactivate GBA2 or GBA3, two other cellular β-glucosidases (13). Specificity for GBA1 generated by the installment of a group at cyclophellitol C-5 inspired Vocadlo and collaborators to design along this principle a sophisticated substrate for selective in situ cell measurement of GBA1 activity (17, 30). Motivated by these findings, we aimed to generate 6-*O*-acyl-4MU-β-glucosides with variable acyl length to see if GBA1 is able to process such artificial substrates and the ability of GBA1 to process related endogenous and exogenous substrates. Our investigations confirmed that these 6-*O*-acyl substrates, accessible through straightforward enzymatic synthesis, can indeed act as substrates for human GBA1. To prevent the potential esterase-mediated degradation of the acyl chain of these substrates the ester bond was successfully replaced by other linkages leading to five new 6-*O*-acyl and alkyl-4MU-β-Glc substrate analogs (6–10). Subsequent investigation revealed that the modified substrate with an ether linkage (9) exhibited excellent kinetics, with the maximum rate of enzymatic cleavage (V_max_) being around 3-fold lower compared to 4MU-β-Glc. Moreover, 9 was found to be hydrolyzed only by GBA1 and not the two other related cellular β-glucosidases GBA2 and GBA3. Thus, 9 emerged as an excellent substrate for assessing reduced GBA1 activity in cell and spleen lysates from patients with GD, and therefore has great potential for diagnostic applications. Of note, we earlier reported that 4MU-β-xyloside is also a specific substrate for GBA1 (14); however, this substrate is hydrolyzed at a 50-fold lower rate than 4MU-β-Glc 1, making substrate 9 a superior choice in this aspect. Our newly designed substrate is not suitable for in situ measurement of GBA1 activity in cells. Firstly, detergents and DMSO are required for the solubility of the substrate, conditions that are not compatible with cell viability. Secondly, released fluorescent 4MU is known to diffuse from lysosomes, hampering the detection of lysosomal activity toward a 4MU-equiped substrate.

6-*O*-acyl modification of glucose occurs naturally and has been particularly observed in plants (25). Plants contain sterols that are chemically distinct from animal cholesterol, with prominent phytosterols being β-sitosterol, campesterol and stigmasterol. Glucosylated forms of phytosterols, also known as steryl glycosides (SG), possess glucose attached at the C-3 hydroxyl of the sterol similar to GlcChol. Subsequent acylation of the sugar moiety at the C-6-hydroxyl yields 6-*O*-acyl-glucosyl-sterols (ASG). High levels of SG and ASG are found in fruits, vegetables (including tomatoes and potatoes) as well as beer and wine (25, 26). Phytosterols are nowadays widely used as food additives aiming to lower plasma LDL cholesterol and reduce cholesterol absorption by stimulation of ABCG5/ABCG8-mediated (phyto)sterol intestinal export (27). Notably, β-sitosterol-β-D-glycoside (BSSG) and β-sitosterol (BSS), abundant in some foods, have been detected in human plasma and tissues (28, 29). Upon chronic high intake, plant sterols have been found to accumulate in the brain (31). Exposure to BSSGs has been hypothesized to underlie the historically high prevalence of the neurodegenerative disease amyotrophic lateral sclerosis–parkinsonism dementia complex (ALS–PDC) on the island of Guam. Indeed, feeding rats with BSSGs causes several neurological signs and defects resembling those occurring in Parkinson’s disease patients, such as α-synuclein aggregates, motor abnormalities, and striatal dopamine loss (32, 33). Van Kampen and co-workers successfully induced parkinsonism in Sprague Dawley rats by feeding them with BSSG for 4 months (34). Interestingly, abnormalities in the *GBA1* gene, encoding GBA1, have been linked to increased risk for Parkinson’s disease and Lewy-body dementia (9, 10).

Our study revealed the presence of glycosylcholesterol and 6-*O*-acyl-glycosyl-cholesterol in spleens, the former being increased in the organs of patients with GD. Surprisingly, plant glycosyl-phytosterols and 6-*O*-acyl derivatives were also detected in the examined spleens. These were elevated in spleens of patients with GD, most strikingly 6-*O*-acyl-glycosyl-β-sitosterol. This finding is consistent with the observation that 4MU-β-Glc and 6-*O*-acyl-4MU-β-Glc serve as substrates for GBA1, the lysosomal glucocerebrosidase deficient in Patients with GD . Our data suggest the potential absorption of glycosylphytosterols and 6-*O*-acyl derivates from plant-derived food. However, we cannot exclude that plant glycosylated sterols enter the body and undergo subsequent acylation (32). Little is so far reported on the kinetics of uptake and metabolism of phytosterols. In a follow-up investigation, we will address this by exposing healthy individuals to glycosylated phytosterol and subsequent assessment of its levels in plasma. Topics for future investigation may be the potential excretion of glycosylated phytosterols by bile or intestine, binding to plasma protein and mechanism of entry into the brain. To the best of our knowledge, our present study offers the first example of increased levels of exogenous plant-derived glycolipids in patients with GD. It prompts the speculation that food may contribute to the increased risk for PD noted for individuals with a mutant *GBA1* allele. Further clinical investigations on this should shed light on this theory. Of note, Akiyama and colleagues earlier reported the presence of plant-type β-sitosterylglucoside in the chicken brain (20).

To conclude, our study shows that human GBA1, deficient in Gaucher disease, can remove 6-*O*-acyl-Glc from 4-methylumbelliferone as well as from cholesterol and plant-derived campesterol and sitosterol. The newly generated substrate 9 (6-*O*-alkyl-4MU-β-Glc), due to its specificity for GBA1, emerges as an attractive tool for diagnostically assessing GBA1 activity in materials that contain the β-glucosidases GBA2 and GBA3.

### Data availability

The authors declare that all data supporting the findings of this study are available within the article and supporting information, and raw data files are available from the corresponding authors upon request.

## Supplemental data

This article contains supplemental data (24, 35, 36, 37, 38).

## Conflict of interest

The authors declare that they have no conflicts of interest with the contents of this article.

## References

[1] Brady R.O., Kanfer J.N., Bradley R.M., Shapiro D. (1966). Demonstration of a deficiency of glucocerebroside-cleaving enzyme in Gaucher's disease. J. Clin. Invest..

[2] Ferraz M.J., Kallemeijn W.W., Mirzaian M., Herrera Moro D., Marques A., Wisse P. (2014). Gaucher disease and Fabry disease: new markers and insights in pathophysiology for two distinct glycosphingolipidoses. Biochim. Biophys. Acta.

[3] Gaucher P.C.E. (1882).

[4] Beutler E., Grabowski G.A., Scriver C.R., Beaudet A.L., Sly W.S., Valle D. (2001). The Metabolic and Molecular Bases of Inherited Disease.

[5] Dardis A., Michelakakis H., Rozenfeld P., Fumic K., Wagner J., Pavan E., International Working Group of Gaucher Disease (IWGGD) (2022). Patient centered guidelines for the laboratory diagnosis of Gaucher disease type 1. Orphanet J. Rare Dis..

[6] Hruska K.S., LaMarca M.E., Scott C.R., Sidransky E. (2008). Gaucher disease: mutation and polymorphism spectrum in the glucocerebrosidase gene (GBA). Hum. Mutat..

[7] Stirnemann J., Vigan M., Hamroun D., Heraoui D., Rossi-Semerano L., Berger M.G. (2012). The French Gaucher's disease registry: clinical characteristics, complications and treatment of 562 patients. Orphanet J. Rare Dis..

[8] Aerts J.M.F.G., Kuo C.L., Lelieveld L.T., Boer D.E.C., van der Lienden M.J.C., Overkleeft H.S. (2019). Glycosphingolipids and lysosomal storage disorders as illustrated by gaucher disease. Curr. Opin. Chem. Biol..

[9] Sidransky E., Nalls M.A., Aasly J.O., Aharon-Peretz J., Annesi G., Barbosa E.R. (2009). Multicenter analysis of glucocerebrosidase mutations in Parkinson's disease. New Engl. J. Med..

[10] Siebert M., Sidransky E., Westbroek W. (2014). Glucocerebrosidase is shaking up the synucleinopathies. Brain.

[11] van Weely S., Brandsma M., Strijland A., Tager J.M., Aerts J.M. (1993). Demonstration of the existence of a second, non-lysosomal glucocerebrosidase that is not deficient in Gaucher disease. Biochim. Biophys. Acta.

[12] Dekker N., Voorn-Brouwer T., Verhoek M., Wennekes T., Narayan R.S., Speijer D. (2011). The cytosolic β-glucosidase GBA3 does not influence type 1 Gaucher disease manifestation. Blood Cell Mol. Dis..

[13] Artola M., Kuo C.L., Lelieveld L.T., Rowland R.J., van der Marel G.A., Codée J.D.C. (2019). Functionalized cyclophellitols are selective glucocerebrosidase inhibitors and induce a bona fide neuropathic gaucher model in zebrafish. J. Am. Chem. Soc..

[14] Boer D.E., Mirzaian M., Ferraz M.J., Zwiers K.C., Baks M.V., Hazeu M.D. (2021). Human glucocerebrosidase mediates formation of xylosyl-cholesterol by β-xylosidase and transxylosidase reactions. J. Lipid Res..

[15] Witte M.D., Kallemeijn W.W., Aten J., Li K.Y., Strijland A., Donker-Koopman W.E. (2010). Ultrasensitive in situ visualization of active glucocerebrosidase molecules. Nat. Chem. Biol..

[16] Kallemeijn W.W., Li K.Y., Witte M.D., Marques A.R., Aten J., Scheij S. (2012). Novel activity-based probes for broad-spectrum profiling of retaining β-exoglucosidases in situ and in vivo. Angew. Chem. (International ed. English).

[17] Yadav A.K., Shen D.L., Shan X., He X., Kermode A.R., Vocadlo D.J. (2015). Fluorescence-quenched substrates for live cell imaging of human glucocerebrosidase activity. J. Am. Chem. Soc..

[18] Akiyama H., Ide M., Nagatsuka Y., Sayano T., Nakanishi E., Uemura N. (2020). Glucocerebrosidases catalyze a transgalactosylation reaction that yields a newly-identified brain sterol metabolite, galactosylated cholesterol. J. Biol. Chem..

[19] Marques A.R., Mirzaian M., Akiyama H., Wisse P., Ferraz M.J., Gaspar P. (2016). Glucosylated cholesterol in mammalian cells and tissues: formation and degradation by multiple cellular β-glucosidases. J. Lipid Res..

[20] Akiyama H., Nakajima K., Itoh Y., Sayano T., Ohashi Y., Yamaguchi Y. (2016). Aglycon diversity of brain sterylglucosides: structure determination of cholesteryl- and sitosterylglucoside. J. Lipid Res..

[21] Aerts J.M., Donker-Koopman W.E., Brul S., Van Weely S., Sa Miranda M.C., Barranger J.A. (1990). Comparative study on glucocerebrosidase in spleens from patients with Gaucher disease. Biochem. J..

[22] Aerts J.M., Sa Miranda M.C., Brouwer-Kelder E.M., Van Weely S., Barranger J.A., Tager J.M. (1990). Conditions affecting the activity of glucocerebrosidase purified from spleens of control subjects and patients with type 1 Gaucher disease. Biochim. Biophys. Acta.

[23] Aerts J.M., Donker-Koopman W.E., van der Vliet M.K., Jonsson L.M., Ginns E.I., Murray G.J. (1985). The occurrence of two immunologically distinguishable beta-glucocerebrosidases in human spleen. Eur. J. Biochem..

[24] Saehlim N., Athipornchai A., Sirion U., Saeeng R. (2020). New class of alkynyl glycoside analogues as tyrosinase inhibitors. Bioorg. Med. Chem. Lett..

[25] Nyström L., Schär A., Lampi A.M. (2012). Steryl glycosides and acylated steryl glycosides in plant foods reflect unique sterol patterns. Eur. J. Lipid Sci. Technol..

[26] Ostlund R.E. (2002). Phytosterols in human nutrition. Annu. Rev. Nutr..

[27] Sabeva N.S., Liu J., Graf G.A. (2009). The ABCG5 ABCG8 sterol transporter and phytosterols: implications for cardiometabolic disease. Curr. Opin. Endocrinol. Diabetes Obes..

[28] Lütjohann D., Björkhem I., Beil U.F., von Bergmann K. (1995). Sterol absorption and sterol balance in phytosterolemia evaluated by deuterium-labeled sterols: effect of sitostanol treatment. J. Lipid Res..

[29] Ostlund R.E., McGill J.B., Zeng C.M., Covey D.F., Stearns J., Stenson W.F. (2002). Gastrointestinal absorption and plasma kinetics of soy Delta(5)-phytosterols and phytostanols in humans. Am. J. Physiol. Endocrinol. Metab..

[30] Deen M.C., Proceviat C., Shan X., Wu L., Shen D.L., Davies G.J. (2020). Selective fluorogenic β-glucocerebrosidase substrates for convenient analysis of enzyme activity in cell and tissue homogenates. ACS Chem. Biol..

[31] Vanmierlo T., Weingärtner O., van der Pol S., Husche C., Kerksiek A., Friedrichs S. (2012). Dietary intake of plant sterols stably increases plant sterol levels in the murine brain. J. Lipid Res..

[32] Shen W.B., McDowell K.A., Siebert A.A., Clark S.M., Dugger N.V., Valentino K.M. (2010). Environmental neurotoxin-induced progressive model of parkinsonism in rats. Ann. Neurol..

[33] Shimamura M. (2020). Structure, metabolism and biological functions of steryl glycosides in mammals. Biochem. J..

[34] Van Kampen J.M., Robertson H.A. (2017). The BSSG rat model of Parkinson's disease: progressing towards a valid, predictive model of disease. EPMA J..

[35] Lelieveld L.T., Mirzaian M., Kuo C.L., Artola M., Ferraz M.J., Peter R.E.A. (2019). Role of β-glucosidase 2 in aberrant glycosphingolipid metabolism: model of glucocerebrosidase deficiency in zebrafish. J. Lipid Res..

[36] Gu Z., Eils R., Schlesner M. (2016). Complex heatmaps reveal patterns and correlations in multidimensional genomic data. Bioinformatics (Oxford, England).

[37] Dussouy C., Téletchéa S., Lambert A., Charlier C., Botez I., De Ceuninck F. (2020). Access to Galectin-3 inhibitors from chemoenzymatic synthons. J. Org. Chem..

[38] Michihata N., Kaneko Y., Kasai Y., Tanigawa K., Hirokane T., Higasa S. (2013). High-yield total synthesis of (-)-strictinin through intramolecular coupling of gallates. J. Org. Chem..

