# Supplementary material for: 6-O-alkyl 4-methylumbelliferyl-β-D-glucosides as selective substrates for GBA1 in the discovery of glycosylated sterols
Source: J Lipid Res. 2024 Oct 10;65(11):100670. doi: 10.1016/j.jlr.2024.100670 (PMC11585764; doi:10.1016/j.jlr.2024.100670)
Supplement: Supplementary information [file mmc1.docx]

**Supplementary information**

**6-*O*-Alkyl 4-methylumbelliferyl-β-D-glucosides as selective substrates for GBA1 in the discovery of glycosylated sterols**

Stef Bannink^1^; Kateryna O. Bila^1^; Joosje van Weperen^1^; Nina A.M. Ligthart^1^; Maria J. Ferraz^1^; Rolf G. Boot^1^; Daan van der Vliet^3^; Daphne. E.C. Boer^1^; Herman S. Overkleeft^2^; Marta Artola^1^*; Johannes M.F.G. Aerts^1^*

^1^Medical Biochemistry, ^2^Bio-organic Synthesis, ^3^Molecular Physiology, Leiden Institute of Chemistry (LIC), Leiden University, 2300 RA Leiden, The Netherlands

Corresponding authors: [j.m.f.g.aerts@lic.leidenuniv.nl](mailto:j.m.f.g.aerts@lic.leidenuniv.nl) and [m.e.artola@lic.leidenuniv.nl](mailto:m.e.artola@lic.leidenuniv.nl)

**TABLE OF CONTENTS**

1. Supporting Figures and Tables S3-9
2. Materials and Methods S10-16
   1. Biochemical and Biological Methods S10
   2. Chemical Synthesis ` S10
      1. General Experimental Details S10-11
      2. Synthesis and Characterization Data of Compounds S11-16
3. Experimental procedures S16-29
4. NMR Spectra S30-57
5. References S58
6. Supporting figures and tables

Table S1. Molecular species, transitions and retention times used to analyze the presence of the (Acyl)HexSterols measured in spleens of non-GD and GD patients.

| **Molecular Species** [M+NH_4_]^+^ | **Transition *m/z*** | **Retention (min)** |
| --- | --- | --- |
| ^13^C_6_-GlcChol | 572.6 > 369.4 | 1.57 |
| HexChol | 566.6 > 369.4 | 1.57 |
| HexCampesterol | 580.4 > 383.4 | 1.70 |
| HexStigmasterol | 592.4 > 395.4 | 1.67 |
| HexSitosterol | 594.5 > 397.4 | 1.79 |
| C-6-*O*-palmitoyl ^13^C_6_-GlcChol | 810.7 > 369.4 | 2.19 |
| AcylHexChol 16:1 | 802.7 > 369.4 | 1.77 |
| AcylHexChol 16:0 | 804.7 > 369.4 | 2.18 |
| AcylHexChol 18:2 | 828.7 > 369.4 | 1.83 |
| AcylHexChol 18:1 | 830.7 > 369.4 | 2.18 |
| AcylHexChol 18:0 | 832.7 > 369.4 | 2.71 |
| AcylHexCampesterol 16:1 | 816.7 > 383.4 | 1.49 |
| AcylHexCampesterol 16:0 | 818.7 > 383.4 | 1.74 |
| AcylHexCampesterol 18:2 | 842.7 > 383.4 | 1.53 |
| AcylHexCampesterol 18:1 | 844.7 > 383.4 | 1.79 |
| AcylHexCampesterol 18:0 | 846.7 > 383.4 | 2.16 |
| AcylHexStigmasterol 16:1 | 828.7 > 395.4 | 1.82 |
| AcylHexStigmasterol 16:0 | 830.7 > 395.4 | 1.74 |
| AcylHexStigmasterol 18:2 | 854.7 > 395.4 | 1.51 |
| AcylHexStigmasterol 18:1 | 856.7 > 395.4 | 1.77 |
| AcylHexStigmasterol 18:0 | 858.7 > 395.4 | 2.13 |
| AcylHexSitosterol 16:1 | 830.7 > 397.4 | 1.60 |
| AcylHexSitosterol 16:0 | 832.7 > 397.4 | 1.88 |
| AcylHexSitosterol 18:2 | 856.7 > 397.4 | 1.63 |
| AcylHexSitosterol 18:1 | 858.7 > 397.4 | 1.90 |
| AcylHexSitosterol 18:0 | 860.7 > 397.4 | 2.29 |

*Footnote: AcylHexCholesterol data was measured on a Waters UPLC-Xevo-QS micro instrument where the C-6-O-palmitoyl 13C6-GlcChol 16:0 (****31****) internal standard showed a retention time of 2.19 min. AcylHexSterols were measured on a second identical machine and we observed a shift in retention times (13C6-palmitoylGlcChol 16:0 ; rt = 1.65 min).*

**Figure S1.** GBA activity measured in lysates (28.1 mg protein/mL) of a non-GD and GD spleen using 4MU-β-Glc, 4MU-β-Xyl and 4MU-C-6-ether **9** as fluorogenic substrates. 4MU-β-Xyl did not show significant 4MU signal (~1 nmol/h/mg protein).

**Figure S2**. Levels (pmol/mg protein) of glycosylcholesterol (HexChol) and sterolins (glycosyl-stigmasterol, glycosyl-β-sitosterol, glycosyl-campesterol) in lysates of spleens (30 mg protein/mL) from non-GD or GD patients determined by LC-MS/MS analysis. ^13^C_6_-GlcChol was used as an internal standard. The red line represents the mean value of the measured samples and the *p*-values are denoted as ns (>0.05), * (≤0.05), ** (≤0.01) and *** (≤0.001).


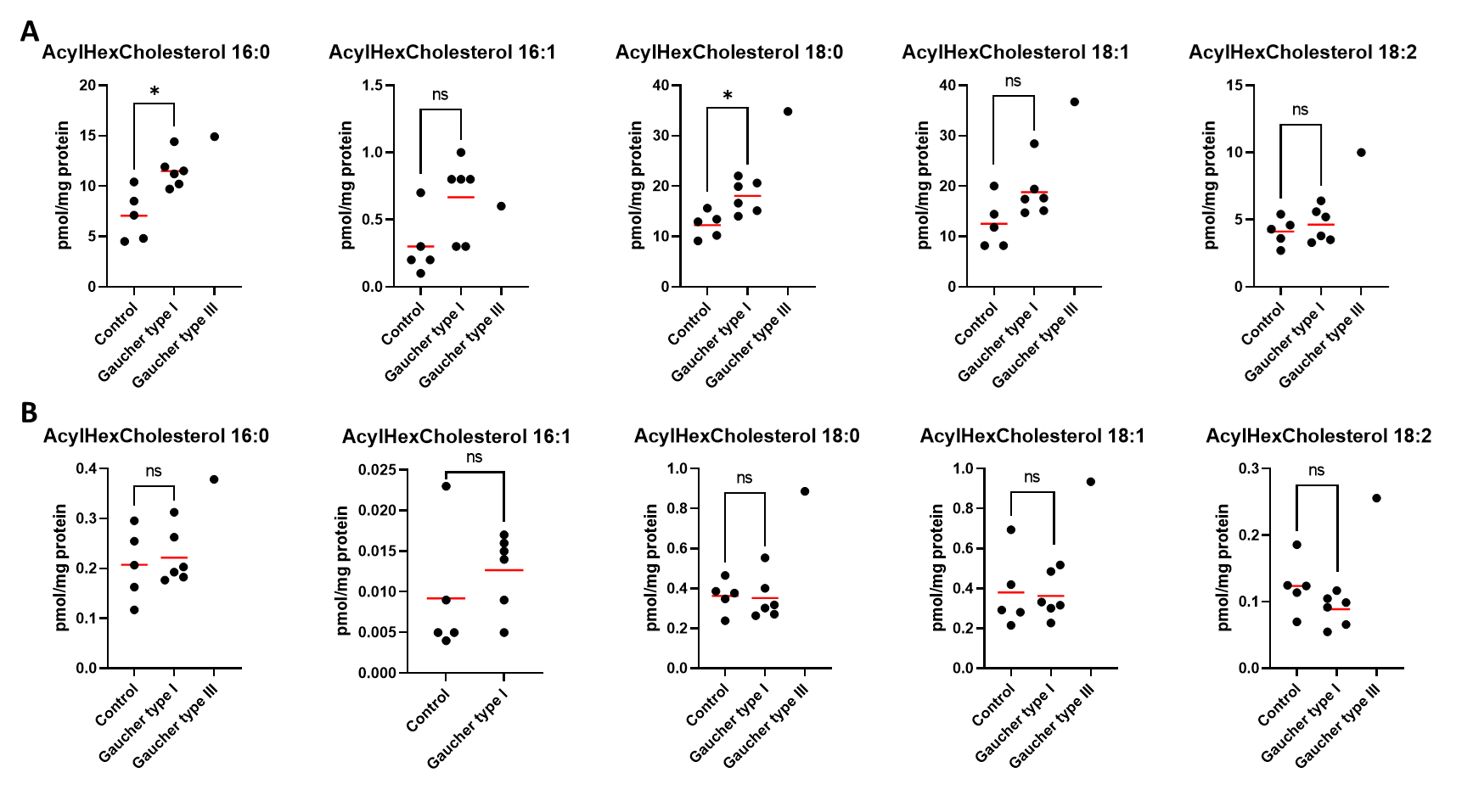


**Figure S3.** Levels (pmol/mg protein) of 6-O-acyl-glycosyl-cholesterol (6-O-palmitoyl and stearoyl versions) in lysates of spleens (30 mg protein/mL) from healthy or GD patients using (A) ^13^C_6_-GlcChol as an internal standard or (B) 6-O-palmitoyl-^13^C_6_-GlcChol (**31**) as an internal standard. The red line represents the mean value of the measured samples and the p-values are denoted as ns (>0.05), * (≤0.05), ** (≤0.01) and *** (≤0.001).


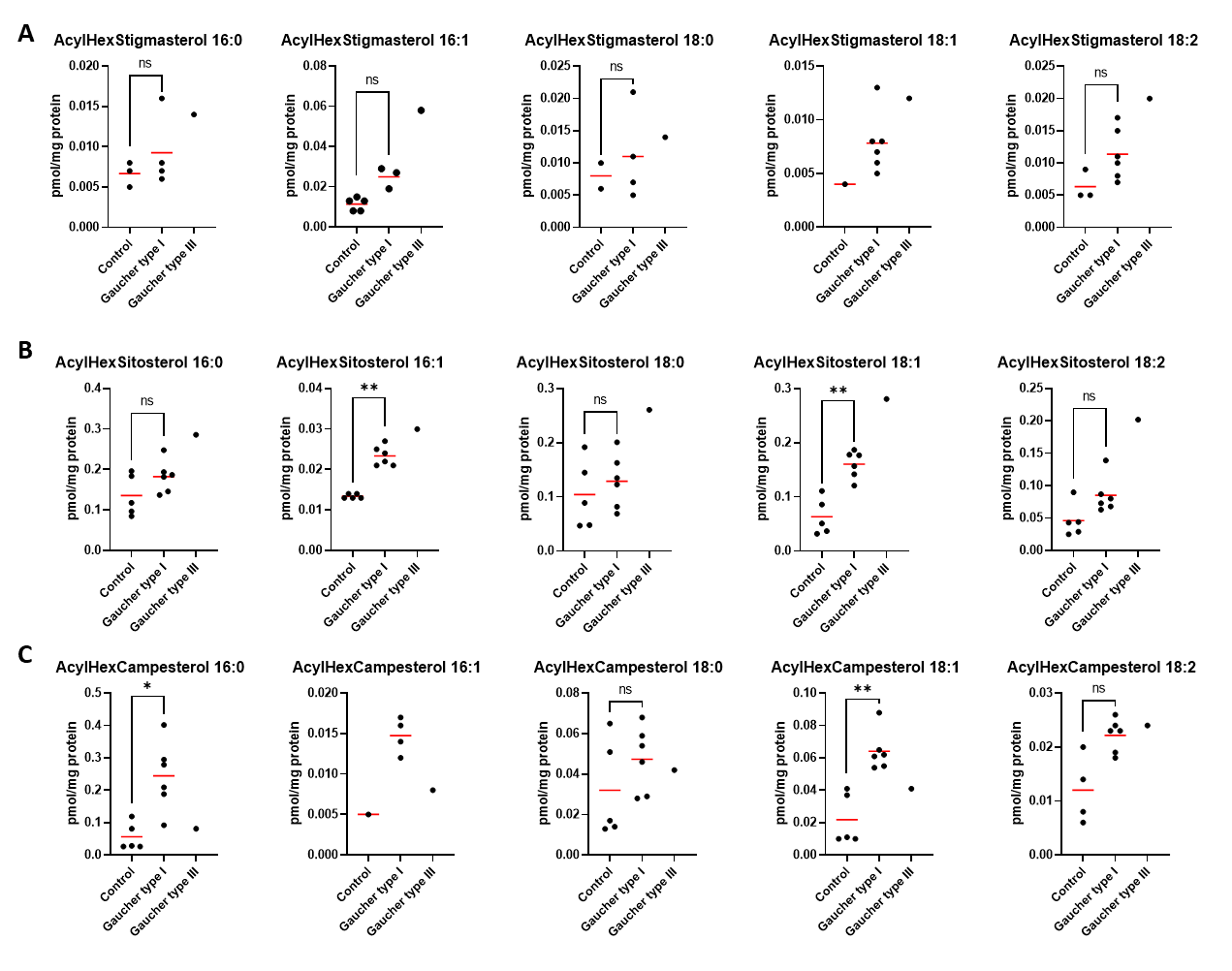


**Figure S4.** Levels (pmol/mg protein) of (A) 6-O-acyl-glycosyl-stigmasterol (AcylHexStigmasterol), (B) 6-O-acyl-glycosyl-β-sitosterol (AcylHexSitosterol) or (C) 6-O-acyl-glycosyl-campesterol (AcylHexCampesterol) in lysates of spleens (30 mg protein/mL) from non-GD or GD patients determined by LC-MS/MS analysis. Data was analyzed using 6-O-palmitoyl-^13^C_6_-GlcChol (**31**) as an internal standard. The red line represents the mean value of the measured samples and the p-values are denoted as ns (>0.05), * (≤0.05), ** (≤0.01) and *** (≤0.001).


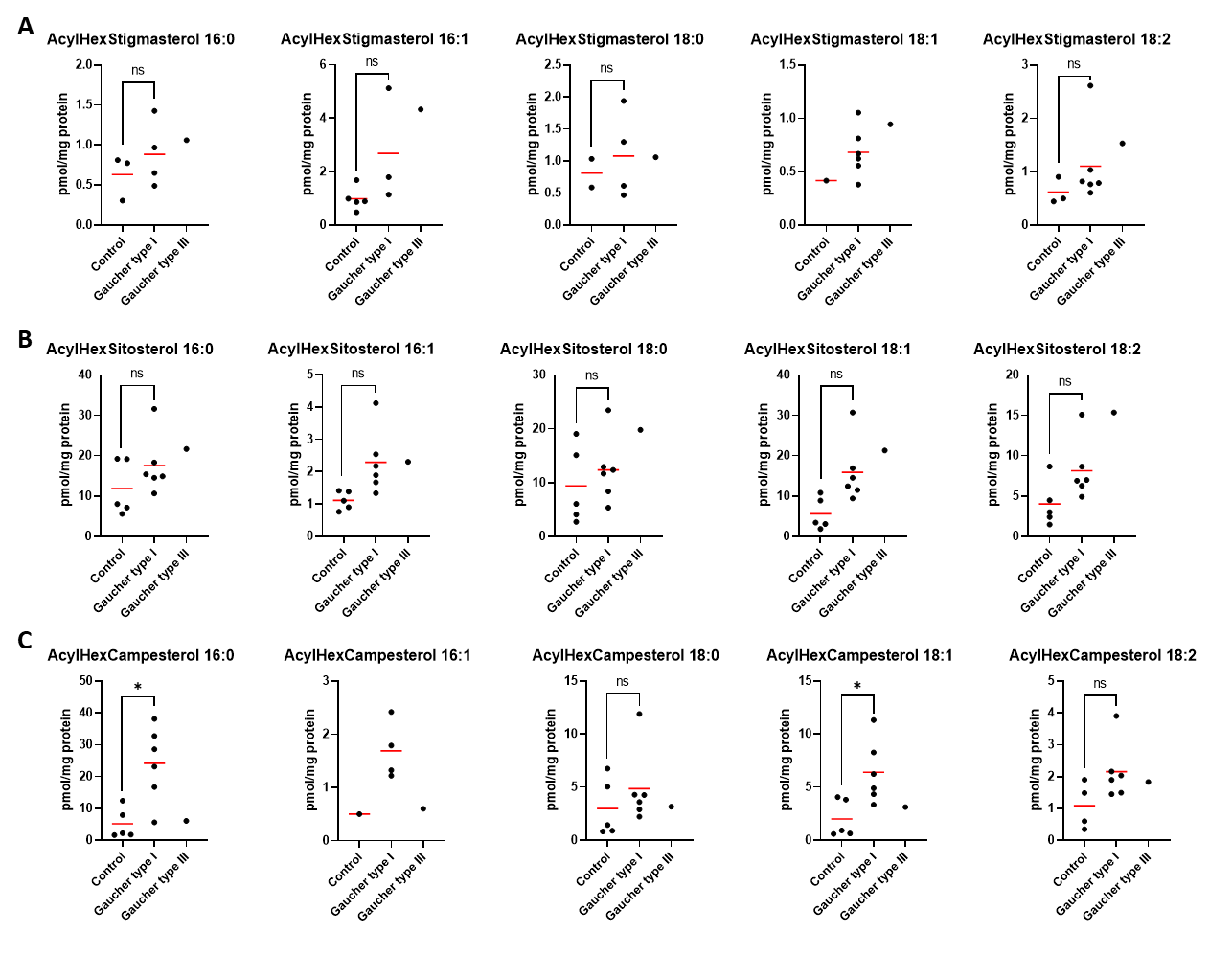


**Figure S5.** Levels (pmol/mg protein) of (A) 6-O-acyl-glycosyl-stigmasterol (AcylHexStigmasterol), (B) 6-O-acyl-glycosyl-β-sitosterol (AcylHexSitosterol) or (C) 6-O-acyl-glycosyl-campesterol (AcylHexCampesterol) in lysates of spleens (30 mg protein/mL) from non-GD or GD patients determined by LC-MS/MS analysis. Data was analyzed using ^13^C_6_-GlcChol as an internal standard. The red line represents the mean value of the measured samples and the p-values are denoted as ns (>0.05), * (≤0.05), ** (≤0.01) and *** (≤0.001).


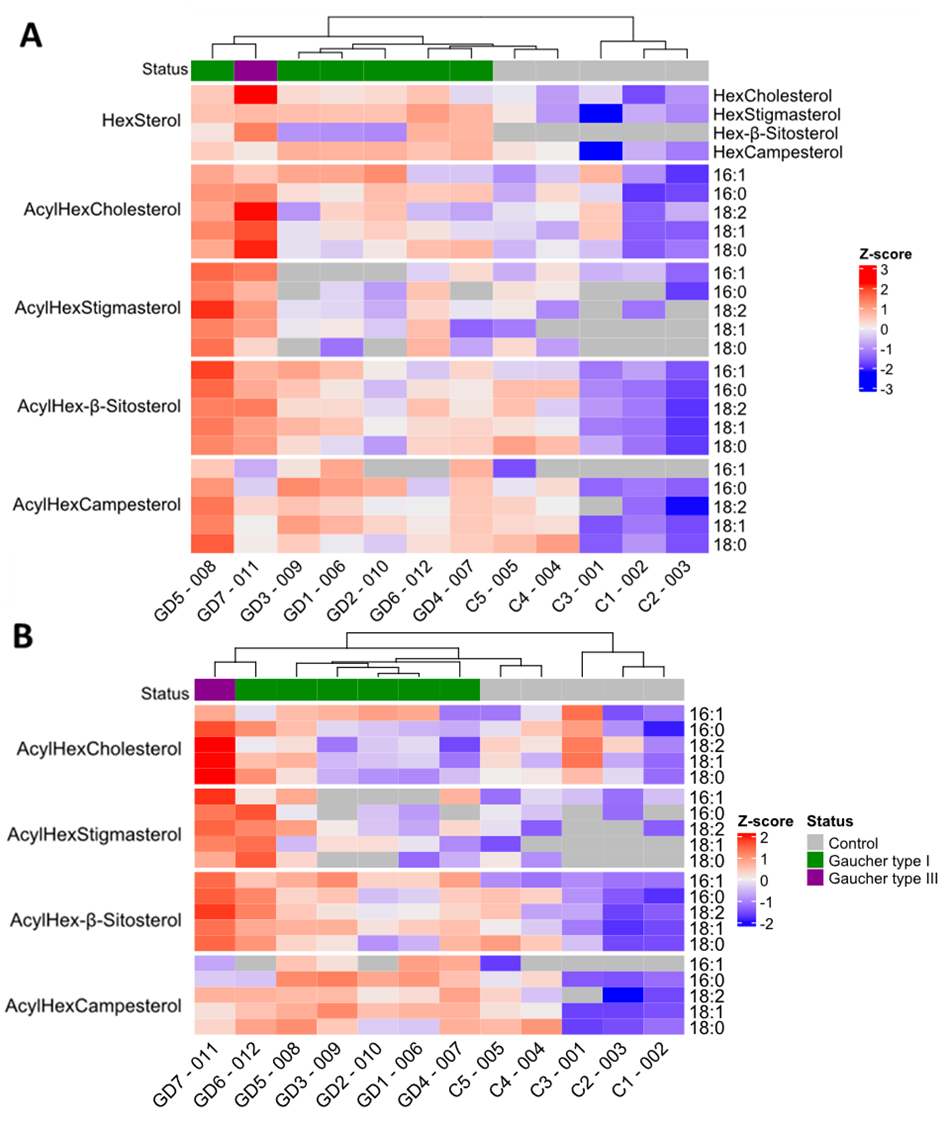


**Figure S6**. Heat map of the levels of glycosylated cholesterol (HexChol) and glycosyl-stigmasterol, glycosyl-β-sitosterol, glycosyl-campesterol and their respective 6-O-acyl forms (16:0, 16:1, 18:0, 18:1 and 18:2) in lysates of spleens (30 mg protein/mL) from control (C1-C5) and GD patients (GD1-GD7) determined by LC-MS/MS analysis using (A) ^13^C_6_-GlcChol or (B) 6-O-palmitoyl-^13^C_6_-GlcChol (**31**) as internal standards. Gaucher type 1, non-neuropathic variant of GD (GD1-GD6); Gaucher type 3 (GD7), sub-acute neuronopathic GD case. This heat map shows the difference observed when using the two different internal standards to determine the amount of lipid accumulation in GD spleens.

**Figure S7**. (A) 100 pmol of C-6-O-acyl-glucosyl-cholesterol before and after deacylation (microwave or 10 h incubation). (B) Formed GlcChol by deacylation of 100 pmol of C-6-O-acyl-glucosyl-cholesterol. (C) C-6-O-acyl-glycosyl-cholesterol (AcylHexChol) and 6-O-acyl-glycosyl-β-sitosterol (AcylHexSito) in spleen sample with and without diacylation. (D) Glycosylated sterols (cholesterol, sitosterol) in GD patient spleen, with and without deacylation. ^13^C_6_-GlcChol was used as an internal standard. Levels are expressed in pmol/mg protein. Samples were extracted as described earlier, followed by deacylation using a microwave-assisted saponification method (36) or incubated in a 37 °C water basin for 10 hours.

**
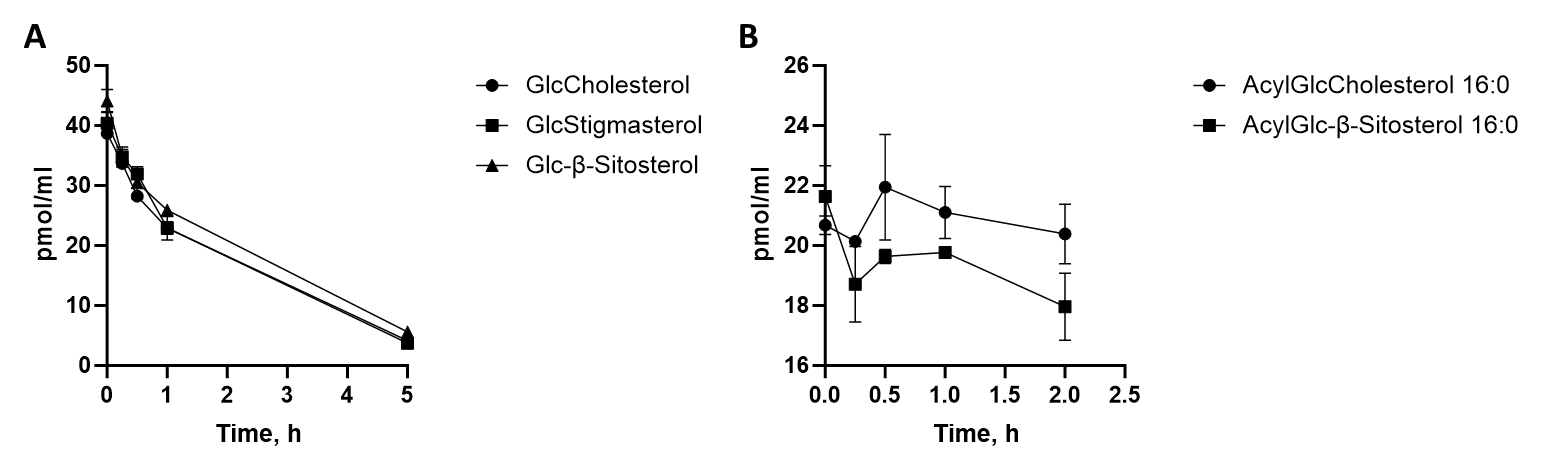
**

**Figure S8**. (A) Degradation of 40 pmol/ml glucosyl cholesterol and glucosyl stigmasterol, β-sitosterol by 272.6 ng/ml rhGBA1 (Cerezyme 1:800x (stock)). Samples of different sterols were incubated for 15 min, 30 min, 1 h and 5 h at 37 ºC in water solution containing 150 mM McIlvaine buffer (pH 5.2), 0.1% BSA, 0.1% Triton X-100, 0.2% sodium taurocholate, 5 % EtOH and (B) 20 pmol/ml 6-O-palmitoyl-glucosyl-cholesterol or 6-O-palmitoyl-glucosyl-β-sitosterol by 272.6 ng/ml rhGBA1 (Cerezyme 1:5000x (final)). Samples of the two acylated sterols were incubated for 15 min, 30 min, 1 h and 2 h at 37 ºC. Data was measured by LC-MS/MS analysis using (A) ^13^C_6_-GlcChol or (B) 6-O-palmitoyl-^13^C_6_-GlcChol as internal standards. Number of technical duplicates n=2.

1. Materials and Methods
   1. Biochemical and Biological Methods

Biochemical and biological studies were performed as described in the main text, and statistical analysis and heatmaps were generated as follows.

**Statistics**

*p*-Values were calculated by a two-sided student’s t-test comparing Gaucher Type I and controls. Lipids for which there were less than 2 measured values per condition were excluded. Raw *p*-values were corrected for multiple testing by the Benjamini-Hochberg method, setting the false discovery rate at 0.05. Lipids for which the adjusted *p*-value was < 0.05 were considered statistically significant. Statistical calculations were performed in R programming software (v4.2.2).

**Heatmap**

To generate the heatmaps, the measured lipid concentrations were log2 transformed and subsequently centered and scaled to unit variance to calculate Z-scores. Heatmaps were generated used the ComplexHeatmap package (v2.14.0) (37) in R programming software (v4.2.2). Hierarchical clustering of samples was performed using Euclidian distances.

- 1. Chemical Synthesis
     1. General Experimental Details

All reagents were of a commercial grade and were used as received unless stated otherwise. Dichloromethane (CH_2_Cl_2_), tetrahydrofuran (THF) and *N*,*N*-dimethylformamide (DMF) were stored over 4 Å molecular sieves, which were dried *in vacuo* before use. Triethylamine and di-isopropyl ethylamine (DIPEA) were dried over KOH and distilled before use. All reactions were performed under an argon atmosphere unless stated otherwise. Solvents used for flash column chromatography were of pro analysis quality. Reactions were monitored by analytical thin-layer chromatography (TLC) using Merck aluminium sheets pre-coated with silica gel 60 with detection by UV absorption (254 nm) and by spraying with a solution of (NH_4_)_6_Mo_7_O_24_·H_2_O (25 g/L) and (NH_4_)_4_Ce(SO_4_)_4_·H_2_O (10 g/L) in 10% sulfuric acid followed by charring at ~150 ˚C or by spraying with an aqueous solution of KMnO_4_ (7%) and K_2_CO_3_ (2%) followed by charring at ~150 °C. Column chromatography was performed manually using either Baker or Screening Device silica gel 60 (0.04 - 0.063 mm) or a Biotage Isolera™ flash purification system using silica gel cartridges (Screening devices SiliaSep HP, particle size 15-40 µm, 60A) in the indicated solvents. ^1^H-NMR and ^13^C-NMR spectra were recorded on Bruker AV-400 (400/100 MHz) and Bruker AV-I-500 (500/125 MHz) spectrometer in the given solvent. Chemical shifts are given in ppm relative to the residual solvent peak used or tetramethylsilane (TMS) as internal standard. Coupling constants are given in Hz. All given ^13^C spectra are proton decoupled. The following abbreviations are used to describe peak patterns when appropriate: s (singlet), d (doublet), t (triplet), qt (quintet), m (multiplet), br (broad), Ar (aromatic), Um (4-methylumbeliferone). 2D-NMR experiments (HSQC, COSY and HMBC) were carried out to assign protons and carbons of the new structures. High-resolution mass spectra (HRMS) of intermediates were recorded with a LTQ Orbitrap (Thermo Finnigan) and final compounds were recorded with an apex-QE instrument (Bruker). LC/MS analysis was performed on an LCQ Advantage Max (Thermo Finnigan) ion-trap spectrometer (ESI+) coupled to a Surveyor HPLC system (Thermo Finnigan) equipped with a C18 column (Gemini, 4.6 mm x 50 mm, 3 μm particle size, Phenomenex) equipped with buffers A: H_2_O, B: acetonitrile (MeCN) and C: 1% aqueous TFA, or an Agilent Technologies 1260 Infinity LCMS with a 6120 Quadrupole MS system equipped with buffers A: H_2_O, B: acetonitrile (MeCN) and C: 100 mM NH_4_OAc. For reversed-phase HPLC-MS purifications an Agilent Technologies 1200 series prep-LCMS with a 6130 Quadrupole MS system was used equipped with buffers A: 50 mM NH_4_HCO_3_ in H_2_O and B: MeCN.

2.2.2. Synthesis and Characterization Data of Compounds

**Scheme S1**. Enzymatic synthesis of 6-O-acyl-4MU-β-Glc substrates **2**-**5** from 4MU-β-Glc with Novozyme 435 and a number of different carboxylic acids. Reagent and conditions: a) i. carboxylic acid (10 eq), dry acetone/pyridine (9:1), 40 °C, 2 h; ii. Novozyme 435 (40 mg/mL, >5000 U/g), 50 °C, 24 h.

The enzymatic esterification of 4-methylumbelliferyl-β-D-glucopyranoside (4MU-β-Glc) was carried out in a mixture of acetone and pyridine in a round-bottom flask (Scheme S1). In particular, 4MU-β-Glc (1 eq) and the desired carboxylic acid (10 eq) were dissolved in dry acetone and pyridine (9:1, volume ratio, 0.059 M). The mixture was heated to 40 °C and stirred for 2 h. Then, Novozyme 435 (40 mg/mL, >5000 U/g) and activated 3Å molecular sieves (160 mg/mL) were added to the mixture and stirred at 50 °C for 24 h under nitrogen atmosphere. The progress of reaction was monitored by thin layer chromatography (CH_2_Cl_2_/MeOH 9:1). After the reaction was completed, the mixture was filtered, the solvents were evaporated and the product was purified by silica gel column chromatography using CH_2_Cl_2_/MeOH (9:1) as eluent to afford the desired acylated 4MU-β-Glc substrates **2**-**5** as white powders.

**Scheme S2**. Synthesis of 6-O-alkyl-4MU-β-Glc linked substrate **9**. Reagent and conditions: a) i. TBSCl, pyridine, 0 °C – r.t., 2 h; ii. BnBr, NaH, DMF, 70 °C, 3 h; iii. HCOOH/H_2_O (4:1), THF, 0 °C to r.t., 3 h, 62% (3 steps). b) 1-bromohexadecane, NaH, DMF, 0 °C to r.t., 16 h, 89%. c) Pd/C, H_2_, HCl, EtOAc/EtOH (1:1), r.t., 4 h, 96%. d) i. H_2_SO_4_, Ac_2_O/AcOH, 0 °C – r.t., 18 h; ii. TMSBr, BiBr_3_, CH_2_Cl_2_, 0 °C to r.t., 18 h; iii. 4MU, NaOH, Acetone/H_2_O (1:1), r.t., dark, 18 h, 36% (3 steps). e) NaOMe, MeOH/CH_2_Cl_2_, r.t., 4 h, 74%.

The synthesis towards the ether-linked substrate **9** started from a-methyl-D-glucose and the initial steps were followed as described in earlier reported synthetic routes towards partially protected intermediate **11** (Scheme S2) (24). Subsequent alkylation of **11** with 1-bromohexadecane using sodium hydride in DMF lead to formation of compound **12** in 89% yield. Benzyl groups were then removed using standard palladium on carbon hydrogenation conditions and intermediate **13** was per-acetylated using sulphuric acid in an acetic acid and acetic anhydride mixture. The crude intermediate was brominated using TMSBr and a catalytic amount of bismuth tribromide. After simple work up, the unstable brominated intermediate was immediately coupled to 4MU in the presence of NaOH to generate intermediate **14** in 37% over 3 steps. Final deacetylation using sodium methoxide lead to the formation of substrate **9** in a total 11% yield over 9 steps.

**Scheme S3**. Synthesis of 6-*O*-amide-4MU-β-Glc **6**. Reagent and conditions: a) i. TrCl, dry pyridine, 80 °C, 18 h; ii. Ac_2_O, 0 °C to r.t., 18 h, 38% (2 steps). b) HBr (33% in AcOH), AcOH, 0 °C, 5 min, 58%. c) TsCl, dry pyridine, 0 °C to r.t., 18 h, 70%. d) NaN_3_, 15-crown-5, DMF, 50 °C, 18 h, 93% (ɑ:β 1:0.4). e) TMSBr, BiBr_3_, CH_2_Cl_2_, 0 °C to r.t., 18 h, 91%. f) 4-MU, NaOH, Acetone/H_2_O (1:1), r.t., dark, 18 h, 62%. g) i. palmitic acid, HOBt, EDC, MeCN/CH_2_Cl_2_, 0 °C, 5 min; ii. **20**, PBu_3_, dark, 5.5 h, 79%. h) NaOMe, MeOH/CH_2_Cl_2_, r.t., 4 h, 77%.

The synthesis towards 6-*O*-amide-4MU-β-Glc substrate **6** started with a one-pot C-6 hydroxyl tritylation and subsequent per-acetylation of D-glucose. The trityl group was then deprotected with 33% HBr in acetic acid to generate intermediate **16**. Tosyl chloride in dry pyridine was used to tosylate the primary hydroxyl, which was reacted with sodium azide and 15-Crown-5 ether to generate azido intermediate **18**. Of note, the basic conditions lead to an epimerization of the anomeric bond. The anomeric position was then brominated using a mixture of TMSBr and bismuth tribromide after which the fluorogenic moiety was coupled using sodium hydroxide in a mixture of acetone and water. Intermediate **21** was treated with palmitic acid in the presence of HOBt, EDC and tributyl phosphine to yield intermediate **20** in 79% yield. This reaction was performed in one-pot to prevent the acetyl migration from C4 to C6, which was observed when using Staudinger ligation conditions. Final deprotection of the acetyl protecting groups then yielded **6** in 0.05% over 9 steps.

*Synthesis of* *6-O-thioester-4MU-β-Glc substrate* ***7*** *and 6-O-thioether-4MU-β-Glc substrate* ***8***

**Scheme S4.** Synthesis of 6-O-thioester-4MU-β-Glc and 6-O-thioether-4MU-β-Glc substrates **7** and **8**, respectively. Reagent and conditions: a) KSAc, DMF, r.t., 18 h, 73%. b) i. HBr (33% in AcOH), CH_2_Cl_2_, 0 °C – r.t., 5 h; ii. 4MU, NaOH, Acetone/H_2_O (1:1), r.t., dark, 18 h, 32% (2 steps). c) NaOMe, MeOH/CH_2_Cl_2_, r.t., 4 h, 78%. d) palmitoyl chloride, Et_3_N, DMF/CH_2_Cl_2_ (2:1), 0 °C – r.t., 6 h, 95% (brsm). e) i. NH_2_NH_2•_H_2_O, AcOH, DMF, r.t., 1 h; ii. 1-bromohexadecane, Et_3_N, CH_2_Cl_2_, 0 °C – r.t., 18 h, 87% (2 steps brsm). f) NaOMe, MeOH/CH_2_Cl_2_, r.t., 4 h, 77%.

Thio-derived 4MU-β-Glc substrates **7** and **8** were synthesized from intermediate **17**. A thioacetate was first installed at C6 by treatment of intermediate **17** with potassium ethanethiolate in DMF. The anomeric position was subsequently brominated with 33% of HBr in acetic acid since bismuth tribromide and TMSBr lead to decomposition of the brominated intermediate. The crude bromide was immediately coupled after a simple work up to 4MU using sodium hydroxide in a mixture of acetone and water. Peracetylated intermediate **23** was then used to generate both products **7** and **8**. Substrate **7** was synthesized by first full deprotection of the acetyl protecting groups using sodium methoxide in methanol followed by selective acylation the thiol with palmityl chloride. Special precautions were taken to mitigate excessive acylation of the secondary hydroxyl groups and unreacted starting material could easily be recovered in this final step leading to a yield of 95% based on recovered starting material. For the synthesis of the alkylated product **8**, the thioacetate in **23** was first deprotected by treatment with hydrazine and subsequently alkylated in the next step without column purification using 1-bromohexadecane and triethylamine to yield intermediate **25** in 87% over 2 steps. Final deprotection using sodium methoxide yielded the desired final compound **8**.

*Synthesis of 6-O-amine-4MU-β-Glc substrate* ***10***

**Scheme S5**. Synthesis of 6-O-amine-4MU-β-Glc substrate **10**. Reagent and conditions: a) i. Ac_2_O, NaOAc, 140 °C, 15 min; ii. PhSH, BF_3_·OEt_2_, CH_2_Cl_2_, 0 °C, 18 h; iii. NaOMe, MeOH/CH_2_Cl_2_, r.t., 4 h; iv. TBSCl, imidazole, DMF, 0 °C – r.t., 18 h; v. Ac_2_O, Et_3_N, DMAP, CH_2_Cl_2_, r.t., 18 h, 68% (5 steps). b) p-TsOH, dry MeOH/CH_2_Cl_2_ (1:1), r.t., 2 h, 89%. c) i. DMP, CH_2_Cl_2_, r.t., 16 h; ii. hexadecan-1-amine, THF, r.t., 2 h; iii. NaCNBH_3_, AcOH, THF, 0 °C – r.t., 18 h, 17% (2 steps). d) i. benzyl chloroformate, DIPEA, THF, 0 °C – r.t., 18 h; ii. Br_2_, CH_2_Cl_2_, dark, 0 °C – r.t., 1.5 h; iii. 4MU, NaOH, TBABr, CHCl_3_/H_2_O (1:1), r.t., dark, 18 h; iv. NaOMe, MeOH/CH_2_Cl_2_, r.t., 4 h; v. Pd/C, H_2_, AcOH, EtOAc/EtOH (1:1), r.t., 1.5 h, 2% (5 steps).

The synthesis towards amine-linked substrate **10** started from D-glucose to generate orthogonally protected intermediate **26** following described procedures (38, 39). The TBS was removed using *para*-toluene sulfonic acid and intermediate **27** was subjected to dess-martin oxidation conditions to generate the aldehyde, which after a simple work up, the crude aldehyde was reacted with 1-aminohexadecane under reductive amination conditions to obtain secondary amine **28**. The low yield of this reaction can be explained by the observed partial deacetylation and migration to the primary alcohol which was difficult to separate from our desired product. A small amount of intermediate **28** was isolated using Prep-HPLC for complete characterization purposes. The secondary amine was then protected using benzyl-chloroformate and DIPEA, and after purification over a short silica-gel plug, the crude was brominated using bromine and subsequently coupled to 4MU using similar conditions as described before but now using a water/chloroform mixture (1:1 v/v) and the addition of TBABr as a phase transfer reagent. This crude intermediate was filtered over a short silica-gel plug and deprotected over 2 steps. First acetyls were removed using sodium methoxide in a mixture of methanol and dichloromethane, and then the Cbz protecting group was removed using palladium on carbon and hydrogen gas. Final column chromatography lead to the isolation of the desired product **10** in a 2% yield over the last five steps. Since the substrate showed no activity in the fluorescent 4MU assay, no further optimization of the synthetic route was pursued.

*Synthesis of 6-O-Palmitoyl-^13^C_6_-Glc-Chol* ***31*** *as an internal standard for the measurement of 6-O-acyl-glucosyl-sterols in GD spleens*

**Scheme S6.** Synthesis of 6-O-palmitoyl-^13^C_6_Glc-Chol. Reagent and conditions: a) i. BzCl, DMAP, pyridine, r.t., 18 h; ii. DMAPA, THF, 0 °C, 3.5 h; iii. TCA, Cs_2_CO_3_, CH_2_Cl_2_, r.t., 18 h, 77% (3 steps). b) Cholesterol, BF_3_·Et_2_O, CH_2_Cl_2_, 0 °C, 3 h, 35% c) i. NaOMe, MeOH/CH_2_Cl_2_, r.t., 4 h ii. Palmitic acid, DIPEA, HATU, pyridine, r.t., 72 h, 27% (2 steps, 88% brsm).

For the measurement of the natural occurrence of 6-*O*-acyl-glucosyl-sterols in non-GD and GD spleens a close structural internal standard of 6-*O*-acyl-glucosyl-sterols was generated for accurate determinations. For this purpose, C-6-*O*-palmitoyl-^13^C_6_Glc-Chol **31** was synthesized as illustrated in Scheme S6. Synthesis started from commercially available ^13^C_6_-D-Glucose which was converted to benzoyl protected imidate donor **29** over three steps. Per-benzoylation with benzoylchloride and catalytic amounts of DMAP in pyridine, followed by deprotection of the anomeric benzoyl group with DMAPA in THF at 0 °C, and final installation of the imidate moiety using standard conditions with trichloroacetonitrile and cesium carbonate as a base yielded imidate **29**. Cholesterol was subsequently glycosylated using **29** and boron trifluoride as an activator to yield benzoyl protected ^13^C_6_-Glc-Chol **30**. Finally, the benzoyl protecting groups were removed using sodium methoxide and C-6 was selectively palmitoylated using palmitic acid, DIPEA and HATU in pyridine to yield **31** in 27% over two steps while recovering a considerable amount of starting material (88% yield brsm over 2 steps).

1. Experimental procedures

**((2*R*,3*S*,4*S*,5*R*,6*S*)-3,4,5-Trihydroxy-6-((4-methyl-2-oxo-2*H*-chromen-7-yl)oxy)tetrahydro-2*H*-pyran-2-yl)methyl hexanoate** **(2)**

Obtained from 4MU-β-Glc (0.2 g, 0.59 mmol) and caprioc acid (687 mg, 5.9 mmol) in dry acetone and pyridine (9:1, volume ratio, 10 mL) in 68% yield (175 mg, 0.40 mmol) following the general procedure described above. ^1^H NMR (500 MHz, CDCl_3_) *δ* 7.36 (d, *J* = 8.7 Hz, 1H, CH-Um), 6.91 (dd, *J* = 8.8, 2.3 Hz, 1H, CH-Um), 6.84 (d, *J* = 2.3 Hz, 1H, CH-Um), 6.07 (d, *J* = 1.3 Hz, 1H, CH-Um), 5.02 (d, *J* = 7.2 Hz, 1H, H-1), 4.40 (d, *J* = 11.4 Hz, 1H, H-6a), 4.28 (dd, *J* = 12.4, 6.7 Hz, 1H, CH, H-6b), 3.74-3.88 (m, 3H, H-2, H-3 and H-5), 3.56 (t, *J* = 8.8 Hz, 1H, H-4), 2.35 – 2.26 (m, 5H, CH_2_-alkyl and CH_3_-Um), 1.58 – 1.42 (m, 2H, CH_2_-alkyl), 1.17 (h, *J* = 3.5 Hz, 4H, 2xCH_2_-alkyl), 0.77 (td, *J* = 6.9, 5.9, 3.1 Hz, 3H, CH_3-_alkyl); ^13^C NMR (126 MHz, CDCl_3_) *δ* 174.5 (CO-acyl), 161.3, 159.8, 154.5, 152.9 (4xC_q_-Um), 125.7 (CH-Um), 114.9 (C_q_-Um), 113.7, 112.6, 104.1 (3xCH-Um), 100.0 (C-1), 76.3 (C-3), 74.2 (C-2), 73.2 (C-5), 70.4 (C-4), 63.4 (C-6), 34.1, 31.2, 24.6, 22.3 (4xCH_2_), 18.6 (CH_3_-Um), 14.0 (CH_3_-alkyl); HRMS: calcd. for C_22_H_28_NaO_9_ [M+Na]^+^ 459.1631, found: 459.1624.

**((2*R*,3*S*,4*S*,5*R*,6*S*)-3,4,5-Trihydroxy-6-((4-methyl-2-oxo-2*H*-chromen-7-yl)oxy)tetrahydro-2*H*-pyran-2-yl)methyl palmitate (3)**

Obtained from 4MU-β-Glc (0.2 g, 0.59 mmol) and palmitic acid (1.52 g, 5.9 mmol) in dry acetone and pyridine (9:1, volume ratio, 10 mL) in 71% yield (243 mg, 0.42 mmol) following the general procedure. ^1^H NMR (500 MHz, CDCl_3_) *δ* 7.44 (d, *J* = 8.7 Hz, 1H, CH-Um), 6.95 (dd, *J* = 8.8, 2.4 Hz, 1H, CH-Um), 6.91 (d, *J* = 2.4 Hz, 1H, CH-Um), 6.12 (d, *J* = 1.3 Hz, 1H, CH-Um), 5.05 – 4.94 (m, 1H, H-1), 4.37 (d, *J* = 4.1 Hz, 2H, H-6a and H-6b), 4.12 (br s, 3H, 3xOH), 3.79 – 3.73 (m, 2H, H-2 and H-3), 3.70 (dt, *J* = 8.9, 4.3 Hz, 1H, H-5), 3.50 (t, *J* = 9.0 Hz, 1H, H-4), 2.39 – 2.31 (m, 5H, CH_2_-alkyl and CH_3_-Um), 1.59 – 1.50 (m, 2H, CH_2_-alkyl), 1.24 (br s, 24H, 12xCH_2_-alkyl), 0.87 (t, *J* = 6.9 Hz, 3H, CH_3_-alkyl). ^13^C NMR (126 MHz, CDCl3) *δ* 174.6 (Cq-alkyl), 161.1, 159.8, 154.8, 152.6 (4xCq-Um), 125.7 (CH-Um), 115.1 (Cq-Um), 113.9, 112.9, 103.7 (3xCH-Um), 99.5 (C-1), 77.3 (C-2/C-3), 74.5 (CH-5), 73.3 (C-2/C-3), 70.2 (C-4), 63.3 (C-6), 35.3, 32.1 (2xCH_2_-alkyl), 29.9 (4xCH_2_-alkyl), 29.8 (2xCH_2_-alkyl), 29.7, 29.5, 29.4, 29.3, 25.0, 23.3 (6xCH_2_-alkyl), 18,9 (CH_3_-Um), 14.3 (CH_3_-alkyl). HRMS: calcd. for C_32_H_49_O_9_ [M+H]^+^ 577.3377, found: 577.3378; HRMS: calcd. for C_32_H_48_NaO_9_ [M+Na]^+^ 599.3196, found: 577.3203.

**((2*R*,3*S*,4*S*,5*R*,6*S*)-3,4,5-Trihydroxy-6-((4-methyl-2-oxo-2*H*-chromen-7-yl)oxy)tetrahydro-2*H*-pyran-2-yl)methyl stearate (4)**

Obtained from 4MU-β-Glc (0.2 g, 0.59 mmol) and stearic acid (1.52 g, 5.9 mmol) in dry acetone and pyridine (9:1, volume ratio, 10 mL) in 60% yield (215 mg, 0.36 mmol) following the general procedure. ^1^H NMR (500 MHz, CDCl_3_) *δ* 7.35 (d, *J* = 8.7 Hz, 1H, CH-Um), 6.90 (d, *J* = 8.7 Hz, 1H, CH-Um), 6.05 (s, 1H, CH-Um), 6.82 (s,1H, CH-Um), 5.18 (br s, 3H, 3OH), 5.00 (d, *J* = 7.0 Hz, 1H, H-1), 4.39 (d, *J* = 11.3 Hz, 1H, H-6a), 4.27 (d, *J* = 5.7 Hz, 1H, H-6b), 3.89 – 3.70 (m, 3H, H-2, H-3 and H-5), 3.55 (t, *J* = 9.0 Hz, 1H, H-4), 2.39 – 2.21 (m, 5H, CH_2_-alkyl and CH_3_-Um), 1.56 – 1.41 (m, 2H, CH_2_-alkyl), 1.34 – 1.04 (m, 28H, 14xCH_2_-alkyl), 0.86 (t, *J* = 7.0 Hz, 3H, CH_3_-alkyl). ^13^C NMR (126 MHz, CDCl_3_) *δ* 174.5 (Cq-alkyl), 161.2, 159.8, 154.5, 152.8 (4xCq-Um), 125.7 (CH-Um), 114.9 (Cq-Um), 113.8, 112.6, 104.1 (3xCH-Um), 100.1 (C-1), 76.3 (C-3), 74.3 (C-5), 73.3 (C-2), 70.5 (C-4), 63.5 (C-6), 34.2, 32.0, (2xCH_2_-alkyl), 29.9 (3xCH_2_-alkyl), 29.9 (3xCH_2_-alkyl), 29.8, 29.7, 29.5, 29.5, 29.3, 25.0, 22.8 (7xCH_2_-alkyl), 18.7 (CH_3_-Um), 14.3(CH_3_-alkyl). HRMS: calcd. for C_34_H_53_O_9_ [M+H]^+^ 605.3690, found: 605.3696; HRMS: calcd. for C_34_H_52_NaO_9_ [M+Na]^+^ 627.3509, found: 627.3517.

**((2*R*,3*S*,4*S*,5*R*,6*S*)-3,4,5-Trihydroxy-6-((4-methyl-2-oxo-2*H*-chromen-7-yl)oxy)tetrahydro-2*H*-pyran-2-yl)methyl oleate (5)**

Obtained from 4MU-β-Glc (0.2 g, 0.59 mmol) and stearic acid (1.52 g, 5.9 mmol) in dry acetone and pyridine (9:1, volume ratio, 10 mL) in 59% yield (210 mg, 0.35 mmol) following the general procedure. ^1^H NMR (500 MHz, CDCl_3_) *δ* 7.34 (d, *J* = 8.8 Hz, 1H, CH-Um), 6.89 (dd, *J* = 8.8, 2.3 Hz, 1H, CH-Um), 6.81 (d, *J* = 2.3 Hz, 1H, CH-Um), 6.03 (s, 1H, CH-Um), 5.40 – 5.20 (m, 2H, CH=CH), 4.97 (d, *J* = 7.1 Hz, 1H, H-1), 4.38 (d, *J* = 11.4 Hz, 1H, H-6a), 4.18 (dd, *J* = 12.0, 7.4 Hz, 1H, H-6b), 3.77 – 3.64 (m, 3H, H2, H3 and H5), 3.47 (t, *J* = 9.1 Hz, 1H, H-4), 2.32 – 2.18 (m, 5H, CH_3_-um and CH_2_-alkyl), 1.99 – 1.90 (m, 4H, 2xCH_2_-alkyl), 1.53 – 1.38 (m, 2H, CH_2_-alkyl), 1.29 – 1.12 (m, 20H, 10xCH_2_-alkyl), 0.84 (t, *J* = 7.0 Hz, 3H, CH_3_-alkyl). ^13^C NMR (126 MHz, CDCl_3_) *δ* 174.5 (Cq-alkyl), 161.3, 159.8, 154.5, 152.9 (4xCq-Um), 130.0, 129.7 (CH=CH), 125.6 (C-Um), 114.8 (Cq-Um), 113.9, 112.5, 104.0 (3xCH-Um), 100.0 (C-1), 76.2, 74.2, 73.2 (C-2, C-3 and C-5), 70.5 (C-4), 63.6 (C-6), 34.1, 32.0 (2xCH_2_-alkyl), 29.8 (2xCH_2_-alkyl), 29.6, 29.5 (2xCH_2_-alkyl), 29.4 (2xCH_2_-alkyl), 29.3, 29.2 (2xCH_2_-alkyl), 27.3 (2xCH_2_-alkyl), 24.9, 22.8 (2xCH_2_-alkyl), 18.6 (CH_3_-Um), 14.2 (CH_3_-alkyl). HRMS: calcd. for C_34_H_51_O_9_ [M+H]^+^ 603.3533, found: 603.3538; HRMS: calcd. for C_34_H_50_NaO_9_ [M+Na]^+^ 625.3353, found: 625.3362.

**((2*R*,3*R*,4*S*,5*R*,6*S*)-3,4,5-Tris(benzyloxy)-6-methoxytetrahydro-2*H*-pyran-2-yl)methanol (11)**

2,3,4-tri-*O*-benzyl-6-hydroxyl-1-methoxyl-α-D-glucopyranoside was synthesized starting from *ɑ*-methyl-D-glucose as described in the literature and its spectroscopic data are in accordance with published data (24).

**(2*R*,3*R*,4*S*,5*R*,6*S*)-3,4,5-Tris(benzyloxy)-2-((hexadecyloxy)methyl)-6-methoxytetrahydro-2*H*-pyran (12)**

A solution of methyl 2,3,4-tri-*O*-benzyl-α-D-glucopyranoside **11** (1.40 g, 3.01 mmol) in DMF (20 mL) and 1-bromohexadecane (1.2 mL, 3.92 mmol)) was added slowly with vigorous stirring to a suspension of NaH (289 mg, 7.23 mmol, 60% in paraffin oil) in DMF (10 mL) at 0 °C. The reaction mixture was stirred for 16 h allowing to reach room temperature. After completion of the reaction, the reaction mixture was quenched with methanol, diluted with diethyl ether and water and extracted. Afterwards the organic layer was washed with brine (3 x 50 mL), dried over MgSO_4_, filtered and concentrated. The crude residue was purified with column chromatography to yield the product (1.85 g, 2.69 mmol, 89 % yield). ^1^H NMR (400 MHz, CDCl_3_) *δ* 7.38 – 7.26 (m, 15H, CH_Ar_), 4.98 (d, *J* = 10.8 Hz, 1H, CH_2_-OBn), 4.89 (d, *J* = 10.9 Hz, 1H, CH_2_-OBn), 4.85 – 4.78 (m, 2H, CH_2_-OBn), 4.67 (d, *J* = 12.2 Hz, 1H, CH_2_-OBn), 4.63 – 4.58 (m, 2H, CH_2_-OBn and H-1), 3.98 (t, 1H, H-3), 3.75 – 3.45 (m, 6H, H-2, H-4, H-5, H-6a and CH_2_-alkyl), 3.37 (s, 3H, CH_3_-OMe), 3.37 – 3.31 (m, 1H, H6b), 1.58 (m, *J* = 6.3 Hz, 2H, CH_2_-alkyl), 1.25 (d, *J* = 9.7 Hz, 26H, 13xCH_2_-alkyl), 0.89 (d, *J* = 13.7 Hz, 3H, CH_3_-alkyl). ; ^13^C NMR (101 MHz, CDCl_3_) *δ* 138.9, 138.6, 138.3 (3xCq-OBn), 128.6, 128.54, 128.52, 128.3, 128.1, 128.0, 127.9, 127.8, 127.7 (15xCH-OBn), 98.3 (C-1), 82.3 (C-3), 80.0, 77.8 (C-2 and C-5), 75.9, 75.2, 73.5 (3xCH_2_-OBn), 71.9 (C-6), 70.1 (C-4), 69.3 (CH_2_-alkyl), 55.3 (CH_3_-OMe), 32.1, 29.83, 29.80, 29.77, 29.75, 29.72, 29.6, 29.5, 26.3, 22.8 (15xCH_2_-alkyl), 14.3 (CH_3_-alkyl). HRMS: calcd. for C_44_H_64_O_6_ [M+NH_4_]^+^ 706.50412, found: 706.50325.

**(2*R*,3*S*,4*S*,5*R*,6*S*)-2-((Hexadecyloxy)methyl)-6-methoxytetrahydro-2*H*-pyran-3,4,5-triol (13)**

Pd/C (85 mg, 0.08 mmol, 10% on charcoal) was added to a stirred solution of the benzyl protected sugar **12** (110 mg, 0.16 mmol) with concentrated HCl (86 µL, 1.05 mmol) in ethanol/ethyl acetate (1/1, v/v, 5 mL). The reaction mixture was then purged with H_2_ and stirred for 4 h at rt. Upon completion, the solid was filtered off and the filtrate was concentrated *in vacuo* and dried to yield the deprotected sugar (64 mg, 0.12 mmol, 96% yield) as a colorless film. ^1^H NMR (400 MHz, CDCl_3_) *δ* 4.74 (d, *J* = 3.6 Hz, 1H, H-1), 3.74 (t, *J* = 9.3 Hz, 1H, H-3), 3.69 – 3.61 (m, 3H, H-4 and CH_2_-alkyl), 3.56 – 3.43 (m, 4H, H-2, H-5, H6a and H6b)), 3.40 (s, 3H, CH_3_-OMe), 1.57 (q, *J* = 7.0 Hz, 2H, CH-alkyl), 1.24 (s, 26H, 13xCH_2_-alkyl), 0.91 – 0.83 (t, 3H, CH_3_-alkyl).; ^13^C NMR (101 MHz, CDCl_3_) *δ* 99.6 (C-1), 74.4 (C-3), 72.3 (C-6), 72.1, 71.1 (C-2 and C-5), 70.5 (C-4), 70.4 (CH_2_-alkyl), 55.3 (CH_3_-OMe), 32.1, 29.85, 29.82, 29.80, 29.74, 29.69, 29.5, 26.2, 22.8 (14xCH_2_-alkyl), 14.3 (CH_3_-alkyl). HRMS: calcd. for C_23_H_46_O_6_ [M+Na]^+^ 441.3187, found: 441.3188.

**(2*R*,3*R*,4*S*,5*R*,6*S*)-2-((Hexadecyloxy)methyl)-6-((4-methyl-2-oxo-2*H*-chromen-7-yl)oxy)tetrahydro-2*H*-pyran-3,4,5-triyl triacetate (14)**

The 2,3,4 deprotected sugar **13** (386 mg, 0.92 mmol) was dissolved in 1:1 AcOH/Ac_2_O (18 mL), and cooled down to 0 °C in an ice bath. Concentrated H_2_SO_4_ (199 µL, 3,55 mmol) was added dropwise into the reaction. The reaction was then removed from the ice bath and stirred at room temperature overnight. After 18 h, the reaction was cooled to 0 °C, and saturated NaHCO_3_ was added dropwise until the reaction was neutralized. The aqueous phase of the reaction was extracted with CH_2_Cl_2_, and the organic layer was recovered, dried over MgSO_4_, filtered, and evaporated to dryness to afford the crude acetylated sugar as a white solid which was used in the next step without further purification. The crude sugar (352 mg, 0.62 mmol) was subsequently dissolved in dry CH_2_Cl_2_ (12 mL) under protected atmosphere. The solution was cooled to 0 °C followed by addition of TMS-Br (0.38 mL, 2.89 mmol) and tribromobismuthane (13.8 mg, 0.03 mmol). The reaction mixture was left stirring overnight allowing to reach room temperature. The reaction mixture was diluted with CH_2_Cl_2_ and washed with sat. aq. NaHCO_3_ and brine. The water layers were extracted with CH_2_Cl_2_ and the combined organic layers were dried (MgSO_4_), filtered and concentrated under reduced pressure. The crude product was used in the next step without further purification. 4MU (90 mg, 0.51 mmol) was added to a solution of NaOH (19.6 mg, 0.49 mmol) in H_2_O (2 mL). To this solution was added the crude sugar bromide (121 mg, 0.20 mmol) dissolved in acetone (2 mL). The mixture was stirred in the dark for 18 h at room temperature. After reaching completion the reaction was diluted with CH_2_Cl_2_ and washed with 1 M NaOH and brine. The water layers were extracted with CH_2_Cl_2_ and the combined organic layers were dried (MgSO_4_), filtered and concentrated under reduced pressure. The product was purified by silica column chromatography (30-50% EtOAc in Pentane) yielding the product (84 mg, 0.12 mmol, 37% yield over 3 steps) as a white solid. ^1^H NMR (400 MHz, CDCl_3_) *δ* 7.53 – 7.47 (m, 1H, CH-Um), 6.97 – 6.91 (m, 2H, 2xCH-Um), 6.18 (d, *J* = 1.3 Hz, 1H, CH-Um), 5.36 – 5.22 (m, 2H, H-2 and H-3), 5.20 – 5.09 (m, 2H, H1 and H-4), 3.85 – 3.77 (m, 1H, H-5), 3.59 (dd, *J* = 10.9, 3.0 Hz, 1H, H-6a), 3.51 (dd, *J* = 10.9, 5.7 Hz, 1H, H-6b), 3.48 – 3.31 (m, 2H, CH_2_-alkyl), 2.39 (s, 3H, CH_3_-Um), 2.05 (d, *J* = 0.8 Hz, 6H, 2xCH_3_-OAc), 2.03 (s, 3H, CH_3_-OAc), 1.58 – 1.47 (m, 2H, CH_2_-alkyl), 1.22 (d, *J* = 13.2 Hz, 26H, 13xCH_2_-alkyl), 0.91 – 0.81 (m, 3H, CH_3_-alkyl).; ^13^C NMR (101 MHz, CDCl_3_) *δ* 170.4, 169.6, 169.4 (3xCq-OAc), 161.5, 159.5, 154.9, 152.3 (4xCq-Um), 125.8 (CH-Um), 115.5 (Cq-Um), 113.9, 113.2, 104.3 (3xCH-Um), 98.5 (C-1), 74.0 (C-5), 72.9 (C-2), 72.3 (CH_2_-alkyl), 71.2 (C-3), 69.5 (C-6), 69.1 (C-4), 32.0, 29.81, 29.77, 29.73, 29.67, 29.6, 29.5, 26.1, 22.8 (14xCH_2_-alkyl), 20.80, 20.76 (3xCH_3_-OAc), 18.8 (CH_3_-Um), 14.2 (CH_3_-alkyl). HRMS: calcd. for C_38_H_56_O_11_ [M+H]^+^ 689.3895, found: 689.3898.

**7-(((2*S*,3*R*,4*S*,5*S*,6*R*)-6-((hexadecyloxy)methyl)-3,4,5-trihydroxytetrahydro-2*H*-pyran-2-yl)oxy)-4-methyl-2*H*-chromen-2-one (9)**

The general procedure for acetyl deprotection with sodium methoxide was followed yielding the final alkylated sugar (51 mg, 0.09 mmol, 74% yield) as a white solid. ^1^H NMR (400 MHz, MeOD/CDCl_3_) *δ* 7.56 (d, *J* = 8.8 Hz, 1H, CH-Um), 7.05 (dd, *J* = 8.8, 2.4 Hz, 1H, CH-Um), 7.01 (d, *J* = 2.4 Hz, 1H, CH-Um), 6.15 (d, *J* = 1.4 Hz, 1H, CH-Um), 4.95 (d, *J* = 7.5 Hz, 1H, H-1), 3.85 – 3.75 (m, 1H, H-3), 3.64 – 3.35 (m, 7H, H-2, H-4, H-5, H-6a, H-6b and CH_2_-alkyl), 2.41 (d, *J* = 1.2 Hz, 3H, CH_3_-Um), 1.60 – 1.47 (m, 2H, CH_2_-alkyl), 1.20 (d, *J* = 20.1 Hz, 26H, 13xCH_2_-alkyl), 0.88 – 0.80 (m, 3H, CH_3_-alkyl). ^13^C NMR (101 MHz, MeOD/CDCl_3_) *δ* 162.6, 160.9, 154.7, 154.1 (4xCq-Um), 126.2 (CH-Um), 115.4 (Cq-Um), 114.3, 112.6, 104.7 (3xCH-Um), 100.4 (C-1), 77.0, 76.2, 73.6 (C-2, C-3 and C-5), 72.4 (CH_2_-alkyl), 70.8 (C-4), 70.4 (C-6), 32.3, 30.1, 30.0, 30.0, 29.9, 29.7, 26.4, 23.0 (14xCH_2_-alkyl), 18.9 (CH_3_-Um), 14.3 (CH_3_-alkyl). HRMS: calcd. for C_32_H_50_O_8_ [M+H]^+^ 563.3578, found: 563.3577.

**(3*R*,4*S*,5*R*,6*R*)-6-((Trityloxy)methyl)tetrahydro-2*H*-pyran-2,3,4,5-tetrayl tetraacetate (15)**

D-Glucose (30 g, 180 mmol) was dissolved in dry pyridine (900 mL) under protected atmosphere. Trityl chloride (50 g, 180 mmol, 1.1 eq) was added and the reaction was left to stir at 80 °C overnight. The reaction was cooled to 0 °C and acetic anhydride (72 mL, 760 mmol, 4.2 eq) was added. The reaction mixture was stirred for 24 h allowing to reach room temperature. The mixture was poured in 5% acetic acid aqueous solution (2.3 L) and stirred for 1 hour. The suspension was filtered and washed with water. The filtrate was resuspended in diethyl ether (200 mL) and filtered once more. The product was recrystallized from warm ethanol giving the product as white solids. (40 g, 68 mmol, 38%).^1^H NMR (400 MHz, CDCl_3_) *δ* 7.46 – 7.19 (m, 15H, Ar-H, OTr), 5.79 – 5.67 (m, 1H, H-1), 5.30 – 5.23 (m, 1H, H-3), 5.22 – 5.15 (m, 2H, H-2 and H-4), 3.69 (ddd, *J* = 9.8, 4.2, 2.5 Hz, 1H, H-5), 3.34 (dd, *J* = 10.6, 2.5 Hz, 1H, H-6a), 3.06 (dd, *J* = 10.7, 4.2 Hz, 1H, H-6b), 2.16 (s, 3H, CH_3_-OAc), 2.04 (s, 3H, CH_3_-OAc), 2.00 (s, 3H, CH_3_-OAc), 1.73 (s, 3H, CH_3_-OAc). ^13^C NMR (101 MHz, CDCl_3_) *δ* 170.4, 169.5, 169.14, 169.09 (4xCq-OAc), 146.6 (3xCq-OTr), 128.9, 127.9, 127.2 (15xCH_Ar_), 92.1 (C‑1), 74.2 (C-5), 73.3 (C-2), 71.8 (C-3), 68.9 (C-4), 61.8 (C-6), 21.0, 20.79, 20.76, 20.6 (4xCH_3_-OAc). HRMS: calcd. for C_33_H_34_O_10_ [M+NH_4_]^+^ 608.2490, found: 608.2487.

**(2*S*,3*R*,4*S*,5*R*,6*R*)-6-(Hydroxymethyl)tetrahydro-2*H*-pyran-2,3,4,5-tetrayl tetraacetate (16)**

The tritylated glucose **15** (11.8 g, 19.98 mmol) was dissolved in glacial acetic acid (70 mL) and cooled to 0 °C. HBr (33% HBr in AcOH solution, 4 mL, 24.31 mmol) was added and the reaction mixture was stirred for 5 minutes and the formed triphenylbromine was immediately filtered off. The solid was washed with water and the water layers were collected extracted with CH_2_Cl_2_ (2 x 80 mL). The combined organic layers were washed with cold water and dried (MgSO_4_), filtered and concentrated under reduced pressure. The crude was purified by silica column chromatography (1:2 EtOAc:Pentane) yielding the product (4.00 g, 11.48 mmol, 58% yield) as a white solid. ^1^H NMR (400 MHz, CDCl_3_) *δ* 5.71 (d, *J* = 8.3 Hz, 1H, H-1), 5.29 (t, *J* = 7,5 Hz, 1H, H-3), 5.17 – 5.00 (m, 2H, H-2 and H-4), 3.75 (d, *J* = 12.7 Hz, 1H, H-6a), 3.63 (ddd, *J* = 9.9, 4.1, 2.1 Hz, 1H, H-5), 3.57 (dd, *J* = 12.6, 4.1 Hz, 1H, H-6b), 2.10 (s, 3H, CH_3_-OAc), 2.06 (s, 3H, CH_3_-OAc), 2.02 (s, 3H, CH_3_-OAc), 2.01 (s, 3H, CH_3_-OAc). ^13^C NMR (101 MHz, CDCl_3_) *δ* 170.4, 170.2, 169.4, 169.2 (4xCq-OAc), 91.8 (C-1), 75.0 (C-5), 72.7 (C-3), 70.5 (C-2), 67.8 (C-4), 60.2 (C-6), 20.9, 20.8, 20.72, 20.69 (4xCH_3_-OAc). HRMS: calcd. for C_14_H_20_O_10_ [M+Na]^+^ 371.09487, found: 371.09442.

**(2*S*,3*R*,4*S*,5*R*,6*R*)-6-((Tosyloxy)methyl)tetrahydro-2*H*-pyran-2,3,4,5-tetrayl tetraacetatese (17)**

1,2,3,4-Tetra-*O*-acetyl-β-D-glucopyranose **16** (3.50 g, 10.1 mmol) was dissolved in dry pyridine (60 mL) under protected atmosphere and cooled 0 °C. Tosyl chloride (2.49 g, 13.1 mmol) was added and the reaction mixture was stirred overnight allowing to reach room temperature. The mixture was diluted with EtOAc and washed with water, 1 M HCl, sat. aq. NaHCO_3_ and Brine. All water layers were extracted with EtOAc and the combined organic layers were dried (MgSO_4_), filtered and concentrated *in vacuo*. The residue was crystallized from warm ethanol giving a white solid (3.54 g, 10.1 mmol, 70%). R_f_ = 0.19 (20% EtOAc in pentane); ^1^H NMR (400 MHz, CDCl_3_) *δ* 7.77 (d, 2 H, *J* = 8.0 Hz, Ar-H-Tosyl), 7.36 (d, 2 H, *J* = 8.0 Hz, Ar-H-Tosyl), 5.65 (d, 1 H, *J* = 8.0 Hz, H-1), 5.20 (t, 1 H, *J* = 9.6 Hz, H-3), 5.09-5.01 (m, 2 H, H-2 and H-4), 4.31 (m, 2 H, H-6), 3.83 (m, 1 H, H-5), 2.46 (s, 3 H, CH_3_-Tosyl), 2.09 (s, 3 H, CH_3_-OAc), 2.04-1.98 (m, 9 H, 3x CH_3_-OAc); ^13^C NMR (101 MHz, CDCl_3_) *δ* 170.2, 169.4, 169.2, 168.9 (4xCq-OAc), 145.2, 132.4 (Cq-Tosyl), 129.9, 128.2 (4xCH-Tosyl), 91.6 (C-1), 72.6 (C-3), 72.2 (C-5), 70.1, 67.9 (C-2 and C-4), 66.8 (C-6), 21.8 (CH_3_-Tosyl, 20.8, 20.61, 20.59, 20.55 (4xCH_3_-OAc). HRMS: calcd. for C_21_H_26_O_12_S [M+NH_4_]^+^ 520.14832, found: 520.14797.

**(3*R*,4*S*,5*R*,6*R*)-6-(Azidomethyl)tetrahydro-2*H*-pyran-2,3,4,5-tetrayl tetraacetate (18)**

Tosylated glucose **17** (3.25 g, 6.47 mmol) was dissolved in dry DMF (60 mL). Sodium azide (0.84 g, 12.9 mmol) and 15-crown-5 (1.41 mL, 7.11 mmol) were added and the reaction was left to stir overnight at 50 °C. The reaction was diluted with EtOAc and extracted with water and brine. The water layers were extracted with EtOAc and the combined organic layers were dried (MgSO_4_), filtered and concentrated *in vacuo*. The product was purified by silica column chromatography (50% EtOAc in pentane to 100% EtOAc) giving a white solid (2.25 g, 6.02 mmol, 93% yield).

ɑ-anomer = ^1^H NMR (CDCl_3_, 400 MHz): *δ* 6.38 (d, 1 H, J = 3.6 Hz, H-1), 5.49 (t, 1H, H-3), 5.13 (t, 1H, J = 9.8 Hz, H-4), 5.12 (dd, 1H, J = 10.4 Hz, H-2), 4.11 (dddd, 1H, H-5), 3.43 (dd, 1H, J = 2.8 Hz , J = 3.6 Hz, H-6a), 3.33 (dd, 1H , J = 5.2 Hz, H-6b), 2.22 (s, 3H, CH_3_-OAc), 2.09 (s, 3H, CH_3_-OAc), 2.06 (s, 3H, CH_3_-OAc), 2.05 (s, 3H, CH_3_-OAc); ^13^C NMR (101 MHz, CDCl_3_):*δ* 170.2, 169.7, 169.5, 168.7 (4xCq-OAc), 88.9 (C-1), 70.9 (C-5), 69.7 (C-3), 69.2, 69.0 (C-2 and C-4), 50.7 (C-6) , 20.9, 20.7, 20.6, 20.5 (4xCH_3_-OAc)).

β-anomer = ^1^H NMR (CDCl_3_, 400 MHz): *δ* 5.75 (1 H, d, H-1), 5.27 (1 H t, H-3), 5.16 (1 H, dd, J = 9.4 Hz, H-2), 5.10 (1 H, t, J = 9.4 Hz, H-4), 3.84 (1 H, ddd, J = 9.4 Hz, H-5), 3.40 (1 H, dd, J = 3.3 Hz, H-6b), 3.37 (1 H, dd, J = 5.3 Hz, H-6a), 2.23 (s, 3H, CH_3_-OAc), 2.09 (s, 3H, CH_3_-OAc), 2.07 (s, 3H, CH_3_-OAc), 2.04 (s, 3H, CH_3_-OAc); ^13^C NMR (101 MHz, CDCl_3_) *δ* 170.1, 169.5, 169.2, 169.0 (4xCq-OAc), 91.5 (C-1), 73.8 (C-5), 72.7 (C-3), 70.1, 69.0 (C-2 and C-4), 50.6 (C-6), 20.8, 20.60, 20.57, 20.55 (4xCH_3_-OAc). HRMS: calcd. for C_14_H_19_O_9_ [M+Na]^+^ 396.10135, found: 396.10064.

**(2*R*,3*R*,4*S*,5*R*,6*R*)-2-(Azidomethyl)-6-bromotetrahydro-2*H*-pyran-3,4,5-triyl triacetate (19)**

6-Azido-glucose **18** (0.37 g, 1.0 mmol, 1 eq) was dissolved in dry CH_2_Cl_2_ (10 mL) under protected atmosphere. The solution was cooled to 0 °C followed by the addition TMSBr (0.52 mL, 4.0 mmol, 4 eq) and BiBr_3_ (22 mg, 0.05 mmol, 0.05 eq). The reaction mixture was left stirring overnight allowing to reach room temperature. The reaction mixture was diluted with CH_2_Cl_2_ and washed with sat. aq. NaHCO_3_ and Brine. The water layers were extracted with CH_2_Cl_2_ and the combined organic layers were dried (MgSO_4_), filtered and concentrated *in vacuo*. The product was purified by silica column chromatography (30% EtOAc in pentane) giving a white solid (0.36 mg, 0.91 mmol, 91%). R_f_ = 0.7 (50% EtOAc in pentane). ^1^H NMR (400 MHz, CDCl_3_) *δ* 6.62 (d, *J* = 4.0 Hz, 1H, H-1), 5.53 (t, *J* = 9.7 Hz, 1H, H-3), 5.18 – 5.09 (m, 1H, H-4), 4.82 (dd, *J* = 10.0, 4.1 Hz, 1H, H-2), 4.29 – 4.22 (m, 1H, H-5), 3.46 (dd, *J* = 13.7, 2.7 Hz, 1H, H-6a), 3.35 (dd, *J* = 13.7, 5.1 Hz, 1H, H-6b), 2.09 (s, 3H, CH_3_-OAc), 2.05 (s, 3H, CH_3_-OAc), 2.03 (s, 3H, CH_3_-OAc).; 13C NMR (101 MHz, CDCl3) *δ* 170.0, 169.9, 169.6 (3xCq-OAc), 86.2 (C-1), 73.1 (C-5), 70.7 (C-2), 70.1 (C-3), 68.4 (C-4), 50.4 (C-6), 30.8 (CH_3_-OAc), 30.71 (CH_3_-OAc), 30.69 (CH_3_-OAc). HRMS: calcd. for C_12_H_16_BrN_3_O_7_ [M+NH_4_]^+^ 411.05099, found 411.05089.

**(2*R*,3*R*,4*S*,5*R*,6*S*)-2-(Azidomethyl)-6-((4-methyl-2-oxo-2*H*-chromen-7-yl)oxy)tetrahydro-2*H*-pyran-3,4,5-triyl triacetate (20)**

4-Methylumbelliferone (0.13 g, 0.75 mmol, 1.5 eq) was added to a solution of NaOH (28 mg, 0.7 mmol, 1.4 eq) in water (1.5 mL). To this solution was added the sugar bromide **19** (0.5 mmol, 1.0 eq) in Acetone (2 mL). The mixture was stirred in the dark overnight at room temperature. The reaction was diluted with CH_2_Cl_2_ and washed with 1 M NaOH and Brine. The water layers were extracted with CH_2_Cl_2_ and the combined organic layers were dried (MgSO_4_), filtered and concentrated *in vacuo*. The product was purified by silica column chromatography (30% to 50% EtOAc in pentane) giving a white solid (152 mg, 0.31 mmol, 62%). R_f_ = 0.4 (50% EtOAc in Pentane). ^1^H NMR (400 MHz, CDCl_3_) *δ* 7.53 (d, J = 8.5 Hz, 1H, CH-um), 6.96 – 6.92 (m, 2H, 2x CH-um), 6.19 (d, J = 1.4 Hz, 1H, CH-um), 5.33 – 5.26 (m, 2H, H-2 and H-3), 5.20 (d, J = 7.5 Hz, 1H, H-1), 5.12 – 5.07 (t, 1H, H-4), 3.83 (ddd, J = 9.8, 6.9, 2.7 Hz, 1H, H-5), 3.43 (dd, J = 13.5, 6.9 Hz, 1H, H-6a), 3.37 (dd, J = 13.5, 2.7 Hz, 1H, H-6b), 2.40 (d, J = 1.4 Hz, 3H, CH_3_-Um), 2.06 (d, J = 2.5 Hz, 6H, 2xCH_3_-OAc), 2.03 (s, 3H, CH_3_-OAc); ^13^C NMR (101 MHz, CDCl_3_) *δ* 170.3, 169.6, 169.3 (3x Cq-OAc) 161.0, 159.1, 154.8, 152.3 (4x Cq-um), 126.0 (CH-um), 115.8 (Cq-um), 113.9, 113.4, 104.3 (3xCH-um), 98.4 (C-1), 73.8, 72.4, 71.0 (C-2, C-3 and C-5), 69.2 (C-4), 51.1 (C-6), 20.72, 20.70 (3xCH_3_-OAc), 18.8 (CH_3_-um); IR (neat) 2100, 1732, 1761, 1612, 1367, 1240 1211, 1066, 1033 cm^-1^. HRMS: calcd. for C_22_H_23_N_3_O_10_ [M+Na]^+^ 512.12756, found: 512.12728.

**(2*S*,3*R*,4*S*,5*R*,6*R*)-2-((4-Methyl-2-oxo-2*H*-chromen-7-yl)oxy)-6-(palmitamidomethyl)tetrahydro-2*H*-pyran-3,4,5-triyl triacetate (21)**

To a solution of palmitic acid (314 mg, 1.23 mmol, 2 eq) and HOBt (188 mg, 1.23 mmol, 2 eq) in acetonitrile (20 mL) and CH_2_Cl_2_ (20 mL) at 0 °C under nitrogen atmosphere was added EDC (235 mg, 1.23 mmol, 2 eq). After stirring for 10 minutes the solution was treated with the 6-azido sugar **20** (300 mg, 0.61 mmol) followed by a toluene solution of tributylphosphine (0.6 M, 0.23 mL, 0.92 mmol, 1.5 eq). The mixture was stirred in the dark for 5.5 h after which it was concentrated under reduced pressure. The crude was purified by silica column chromatography (12:8 EtOAc:Pentane) yielding the pure product as a white amorphous solid (341 mg, 0.49 mmol, 79 % yield). ^1^H NMR (400 MHz, CDCl_3_) *δ* 7.52 (d, *J* = 9.2 Hz, 1H, CH-Um), 6.90 (d, *J* = 8.3 Hz, 2H, 2xCH-Um), 6.19 (d, *J* = 1.3 Hz, 1H, CH-Um), 5.78 (t, *J* = 6.2 Hz, 1H, NH), 5.32 – 5.23 (m, 2H, H-2 and H-3), 5.17 (d, *J* = 7.6 Hz, 1H, H-1), 5.01 (t, *J* = 9.6 Hz, 1H, H-4), 3.86 – 3.78 (m, H-5) 3.61 (ddd, *J* = 14.5, 5.7, 2.8 Hz, 1H, H-6a), 3.49 (dt, *J* = 14.6, 6.4 Hz, 1H, H-6b), 2.40 (d, *J* = 1.3 Hz, 3H, CH_3_-um), 2.22 – 2.16 (m, 2H, CH_2_-alkyl), 2.08 (s, 3H, CH_3_-OAc), 2.06 (s, 3H, CH_3_-OAc), 2.03 (s, 3H, CH_3_-OAc), 1.59 (s, 2H, CH_2_-alkyl), 1.24 (d, *J* = 5.9 Hz, 24H, 12xCH_2_-alkyl), 0.89 – 0.84 (t, 3H, CH_3_-alkyl); ^13^C NMR (101 MHz, CDCl_3_) *δ* 173.5 (Cq-alkyl, 170.8, 169.8, 169.4 (3xCq-OAc), 161.4, 159.2, 155.5, 151.5 (4xCq-Um), 126.5 (CH-Um), 115.0 (Cq-Um), 113.6, 113.5, 104.9 (3xCH-Um), 97.4 (C-1), 73.7 (C-5), 72.6, 71.1 (C-2 and C-3), 68.7 (C-4), 39.1 (C-6), 36.8, 32.1, 29.83, 29.79, 29.7, 29.51, 29.49, 29.4, 25.7, 22.8 (14xCH_2_-alkyl), 20.81, 20.75 (3xCH_3_-OAc), 17.9 (CH_3_-Um), 14.3 (CH_3_-alkyl). HRMS: calcd. for C_38_H_55_N_3_O_11_ [M+H]^+^ 702.38479, found: 702.38445.

***N*-(((2*R*,3*S*,4*S*,5*R*,6*S*)-3,4,5-trihydroxy-6-((4-methyl-2-oxo-2*H*-chromen-7-yl)oxy)tetrahydro-2*H*-pyran-2-yl)methyl)palmitamide (6)**

The protected sugar **21** (30 mg, 0.043 mmol) was dissolved in MeOH (3 mL) followed by the addition of sodium methoxide (30% in MeOH solution, 3 drops). The mixture was stirred for 4 h at room temperature after which TLC indicated complete conversion. The reaction was quenched with amberlite after which it was filtered and washed with MeOH. The solution was concentrated yielding the crude product which was purified by silica column chromatography (5% MeOH in CH_2_Cl_2_) yielding the desired product (17 mg, 0.030 mmol, 77 % yield) as a white solid. ^1^H NMR (400 MHz, MeOD) *δ* 7.64 – 7.57 (d, 1H, CH-Um), 7.03 (dd, *J* = 8.8, 2.4 Hz, 1H, CH-Um), 7.00 (d, *J* = 2.4 Hz, 1H, CH-Um), 6.17 (d, *J* = 1.4 Hz, 1H, CH-Um), 5.02 – 4.94 (m, 1H, H-1), 3.64 – 3.46 (m, 5H, H-2, H-3, H-4, H-6a and H-6b), 3.28 – 3.18 (m, 1H, H-5), 2.43 (d, *J* = 1.2 Hz, 3H, CH_3_-Um), 2.20 (td, *J* = 7.4, 2.1 Hz, 2H, CH_2_-alkyl), 1.60 – 1.50 (m, 2H, CH_2_-alkyl), 1.22 (d, *J* = 8.0 Hz, 24H, 12xCH_2_-alkyl), 0.88 – 0.82 (t, 3H, CH_3_-alkyl). ^13^C NMR (101 MHz, MeOD) *δ* 176.05 (Cq-alkyl), 162.8, 161.1, 155.3, 154.4 (4xCq-Um), 126.5 (CH-Um), 115.6 (Cq-um), 114.6, 112.7, 105.5 (3xCH-Um), 101.1 (C-1), 76.4, 75.6, 74.0 (C-2, C-3 and C-4), 71.7 (C-5), 40.2 (C-6), 36.2, 32.9, 30.24, 30.21, 30.1, 29.94, 29.92, 29.86, 25.6, 23.8 (14xCH_2_-alkyl), 18.9 (CH_3_-Um), 14.3 (CH_3_-alkyl). HRMS: calcd. for C_32_H_49_NO_8_ [M+H]^+^ 576.3531, found: 523.3529.

**(2*S*,3*R*,4*S*,5*S*,6*S*)-6-((Acetylthio)methyl)tetrahydro-2*H*-pyran-2,3,4,5-tetrayl tetraacetate (22)**

Tosylated glucose **17** (2.08 g, 4.12 mmol) was dissolved in dry DMF (50 mL) and potassium ethanethioate (1.41 g, 12.4 mmol, 3 eq) was added at room temperature under nitrogen atmosphere. The reaction mixture was stirred overnight after which it was diluted with EtOAc and extracted with NaHCO_3_, water and brine. The water layers were extracted with EtOAc and the combined organic layers were dried (MgSO_4_), filtered and concentrated under reduced pressure. The product was purified by silica column chromatography (EtOAc:Pentane 6:14) yielding the product (1.22 g, 3.00 mmol, 73% yield) as a white solid. ^1^H NMR (400 MHz, CDCl_3_) *δ* 5.66 (d, *J* = 8.3 Hz, 1H, H-1), 5.21 (t, *J* = 9.4 Hz, 1H, H-3), 5.14 – 5.06 (t, 1H, H-2), 5.06 – 4.98 (t, 1H, H-4), 3.83 – 3.76 (ddd, 1H, H-5), 3.21 (dd, *J* = 14.5, 3.1 Hz, 1H, H-6a), 3.14 (dd, *J* = 14.4, 6.0 Hz, 1H, H-6b), 2.33 (s, 3H CH_3_-SAc), 2.11 (s, 3H CH_3_-OAc), 2.08 (s, 3H CH_3_-OAc), 2.02 (s, 3H CH_3_-OAc), 2.00 (s, 3H, CH_3_-OAc).; ^13^C NMR (101 MHz, CDCl_3_) *δ* 195.4 (Cq-SAc), 170.2, 169.8, 169.3, 169.0 (4xCq-OAc), 91.0 (C-1), 75.0 (C-5), 72.8 (C-3), 70.3 (C-2), 69.8 (C-4), 30.5 (CH_3_-SAc), 29.1 (C-6), 20.9, 20.8, 20.7, 20.6 (4xCH_3_-OAc). HRMS: calcd. for C_16_H_22_O_10_S [M+Na]^+^ 429.08259, found: 429.08205.

**(2*S*,3*S*,4*S*,5*R*,6*S*)-2-((Acetylthio)methyl)-6-((4-methyl-2-oxo-2*H*-chromen-7-yl)oxy)tetrahydro-2*H*-pyran-3,4,5-triyl triacetate (23)**

The acetyl protected sugar **22** (208 mg, 0.51 mmol) was dissolved in CH_2_Cl_2_ (2.5 mL) and cooled to 0 °C. HBr (33% in AcOH, 1.21 mL, 22.3 mmol, 10.8 eq) was added dropwise and the reaction mixture was stirred in the dark for 5 h. The mixture was diluted with CH_2_Cl_2_ and washed with ice water (3x 50 mL), NaHCO_3_ (50 mL) and water again (50 mL). The water layers were extracted with CH_2_Cl_2_ and the combined organic layers were dried (MgSO_4_), filtered and concentrated under reduced pressure. The crude (160 mg, 0.37 mmol, 73% yield) was used in the next step without further purification. 4MU (114 mg, 0.65 mmol) was added to a solution of NaOH (24.9 mg, 0.62 mmol) in H_2_O (2.15 mL). The crude bromo sugar (111 mg, 0.26 mmol) was added to this solution dissolved in acetone (2.8 mL). The mixture was stirred in the dark for 18 h at room temperature. After reaching completion the reaction was diluted with CH_2_Cl_2_ and washed with 1 M NaOH and brine. The water layers were extracted with CH_2_Cl_2_ and the combined organic layers were dried (MgSO_4_), filtered and concentrated under reduced pressure. The product was purified by silica column chromatography (30-50% EtOAc in Pentane) yielding the product (60 mg, 0.114 mmol, 32% yield over 2 steps) as a white solid. ^1^H NMR (400 MHz, CDCl_3_) *δ* 7.51 (d, *J* = 8.7 Hz, 1H, CH-Um), 6.95 (d, 1H, CH-Um), 6.91 (dd, *J* = 8.7, 2.5 Hz, 1H, CH-Um), 6.18 (d, *J* = 1.3 Hz, 1H, CH-Um), 5.32 – 5.21 (m, 2H, H-1, H-3), 5.14 – 5.01 (m, 2H, H-2 and H-4), 3.79 (ddd, *J* = 10.1, 7.7, 2.8 Hz, 1H, H-5), 3.29 (dd, *J* = 14.4, 2.8 Hz, 1H, H-6a), 3.03 (dd, *J* = 14.4, 7.7 Hz, 1H, H-6b), 2.40 (d, *J* = 1.2 Hz, 3H, CH_3_-Um), 2.38 (s, 3H, CH_3_-SAc), 2.10 (s, 3H, CH_3_-OAc), 2.04 (s, 3H, CH_3_-OAc), 2.02 (s, 3H, CH_3_-OAc). ^13^C NMR (101 MHz, CDCl_3_) *δ* 195.3 (Cq-SAc), 170.2, 169.9, 169.4 (3xCq-OAc), 160.9, 159.3, 154.9, 152.3 (4xCq-Um), 124.4 (CH-Um), 115.6 (Cq-Um), 114.0, 112.4, 104.2 (3xCH-Um), 99.6 (C-1), 73.8 (C-5), 72.6, 71.1, 70.5 (C-2, C-3 and C-4), 30.6 (CH_3_-SAc), 30.1 (C-6), 20.8, 20.71, 20.70 (3xCH_3_-OAc), 18.8 (CH_3_-Um). HRMS: calcd. for C_24_H_26_O_11_S [M+H]^+^ 523.1269, found: 523.1269.

**4-Methyl-7-(((2*S*,3*R*,4*S*,5*S*,6*S*)-3,4,5-trihydroxy-6-(mercaptomethyl)tetrahydro-2*H*-pyran-2-yl)oxy)-2*H*-chromen-2-one (24)**

The acetylated thiosugar **23** (60 mg, 0.114 mmol) was dissolved in MeOH (3 mL) followed by the addition of sodium methoxide (30% in MeOH solution, 3 drops). The mixture was stirred for 4 h at room temperature after which TLC indicated complete conversion. The reaction was quenched with amberlite after which it was filtered and washed with MeOH. The solution was concentrated yielding the crude product which was purified by silica column chromatography (5% MeOH in CH_2_Cl_2_) yielding the desired product (50 mg, 0.14 mmol, 78% yield). ^1^H NMR (400 MHz, MeOD) *δ* 7.70 (d, *J* = 8.8 Hz, 1H, CH-Um), 7.13 (dd, *J* = 8.8, 2.5 Hz, 1H, CH-Um), 7.09 (d, *J* = 2.4 Hz, 1H, CH-Um), 6.20 (d, *J* = 1.3 Hz, 1H, CH-Um), 5.10 – 5.01 (m, 1H, H-1), 3.58 – 3.44 (m, 3H, H-2, H-4 and H-5), 3.35 (dd, *J* = 9.4, 4.5 Hz, 1H, H-3), 3.01 (dd, *J* = 14.1, 2.4 Hz, 1H, H-6a), 2.66 (dd, *J* = 14.1, 8.1 Hz, 1H, H-6b), 2.45 (d, *J* = 1.2 Hz, 3H).; ^13^C NMR (101 MHz, MeOD) *δ* 163.3, 161.9, 156.0, 155.4 (4xCq-Um), 127.3 (CH-um), 116.1 (Cq-Um), 114.9, 112.9, 105.0 (3xCH-Um), 101.2 (C-1), 78.7, 77.6, 74.8 (C-2, C-4 and C-5), 73.8 (C-3), 26.9 (C-6), 18.7 (CH_3_-Um). HRMS: calcd. for C_16_H_18_O_7_S [M+H]^+^ 355.0846, found: 355.0846.

***S*-(((2*S*,3*S*,4*S*,5*R*,6*S*)-3,4,5-Trihydroxy-6-((4-methyl-2-oxo-2*H*-chromen-7-yl)oxy)tetrahydro-2*H*-pyran-2-yl)methyl) hexadecanethioate (7)**

The deacetylated thio-sugar **24** (89 mg, 0.25 mmol) was dissolved in dry CH_2_Cl_2_ (1 mL) and DMF (2 mL) and was cooled to 0 °C. To this triethylamine (42 μL, 0.30 mmol, 1.2 eq) and palmitoyl chloride (91 μL, 0.30 mmol, 1.2 eq) were added. The reaction was allowed to warm up to room temperature and was stirred for 6 h after which the solvent was evaporated. The crude was purified on silica column chromatography (5% MeOH in CH_2_Cl_2_) and the residual starting material was recovered yielding the product (64 mg, 0.11 mmol, 43% yield, 95% brsm) as a white solid. ^1^H NMR (400 MHz, MeOD/CDCl_3_) *δ* 7.57 (d, *J* = 9.3 Hz, 1H, CH-Um), 7.02 (m, *J* = 7.8 Hz, 2H, 2xCH-Um), 6.16 (d, *J* = 1.3 Hz, 1H, CH-Um), 4.89 (d, *J* = 7.6 Hz, 1H, H-1), 3.60 – 3.42 (m, 4H, H-2, H-4, H-5, H-6a), 3.35 – 3.26 (m, 1H, H-3), 2.99 (dd, *J* = 14.0, 8.7 Hz, 1H, H-6b), 2.64 – 2.49 (m, 2H, CH_2_-alkyl), 2.42 (d, *J* = 1.2 Hz, 3H, CH_3_-Um), 1.64 – 1.51 (m, 2H, CH_2_-alkyl), 1.30 – 1.16 (m, 24H, 12xCH_2_-alkyl), 0.88 – 0.79 (m, 3H, CH_3_-alkyl). ^13^C NMR (101 MHz, MeOD/CDCl_3_) *δ* 198.9 (Cq-SAc), 162.8, 160.7, 155.4, 153.6 (Cq-Um), 125.7 (CH-Um), 114.9 (Cq-Um), 113.9, 112.2, 104.2 (CH-Um), 100.4 (C-1), 76.1, 75.5, 73.2 (C-2, C-4 and C-5), 72.9 (C-3), 43.2, 31.8, 30.3, 29.6, 29.5 (5xCH_2_-alkyl), 29.5 (C-6), 29.31, 29.25, 29.2, 28.8, 25.5, 22.6 (9xCH_2_-alkyl), 19.6 (CH_3_-Um), 13.8 (CH_3_-alkyl). HRMS: calcd. for C_32_H_48_O_8_S [M+H]^+^ 593.3143, found: 593.3142.

**(2*S*,3*S*,4*S*,5*R*,6*S*)-2-((Hexadecylthio)methyl)-6-((4-methyl-2-oxo-2*H*-chromen-7-yl)oxy)tetrahydro-2*H*-pyran-3,4,5-triyl triacetate (25)**

To a solution of the 6-thio sugar **23** (50 mg, 0.10 mmol) in DMF (2 mL) were added AcOH (6.57 µL, 0.12 mmol) and hydrazine hydrate (3.60 µL, 0.12 mmol), after stirring the reaction mixture for 1 h, the solution was concentrated to halve the original volume. The resulting crude was diluted with EtOAc and washed with water brine. The organic layer was dried with MgSO_4_, filtered and concentrated *in vacuo*. The crude product was used in the next step without further purification. The crude intermediate (46 mg, 0.10 mmol) was dissolved in dry CH_2_Cl_2_ (1 mL) and cooled to 0 °C. To this solution, triethylamine (27 μl, 0.19 mmol) and 1-bromohexadecane (146 μL, 0.48 mmol) were added. The reaction was allowed to warm up to room temperature and was stirred for 18 h. The reaction was quenched with methanol and concentrated under reduced pressure. The crude was purified on silica column chromatography (6:14 EtOAc:pentane) and the residual starting material was recovered yielding the product (14 mg, 0.02 mmol, 21% yield, 87% brsm) as a white solid. ^1^H NMR (400 MHz, CDCl_3_) *δ* 7.52 (d, *J* = 9.1 Hz, 1H, CH-Um), 7.04 – 6.94 (m, 2H, 2xCH-Um), 6.19 (d, *J* = 1.3 Hz, 1H, CH-Um), 5.32 – 5.25 (m, 2H, H-2 and H-3), 5.18 – 5.11 (m, 1H, H-1), 5.11 – 5.04 (m, 1H, H-4), 3.80 (ddd, *J* = 9.8, 8.4, 2.9 Hz, 1H, H-5), 2.72 (dd, *J* = 14.4, 2.9 Hz, 1H, H-6a), 2.62 (dd, *J* = 14.4, 8.4 Hz, 1H, H-6b), 2.51 (t, *J* = 7.5 Hz, 2H, CH_2_-alkyl), 2.40 (d, *J* = 1.2 Hz, 3H, CH_3_-Um), 2.07 (s, 3H, CH_3_-OAc), 2.06 (s, 3H, CH_3_-OAc), 2.03 (s, 3H, CH_3_-OAc), 1.55 – 1.43 (m, 2H, CH_2_-alkyl), 1.23 (d, *J* = 9.2 Hz, 26H, 13xCH_3_-OAc), 0.91 – 0.81 (m, 3H, CH_3_-alkyl). ^13^C NMR (101 MHz, CDCl_3_) *δ* 170.4, 169.7, 169.5 (3xCq-OAc), 161.0, 159.5, 155.0, 152.2 (4xCq-Um), 125.9 (CH-Um), 115.7 (Cq-Um), 113.7, 113.4, 104.5 (3xCH-Um), 98.5 (C-1), 75.9 (C-5), 72.7 (C-3), 71.4 (C-4), 71.2 (C-2), 33.9 (CH_2_-alkyl), 33.2 (C-6), 32.1, 29.9, 29.80, 29.75, 29.68, 29.66, 29.4, 29.0, 22.8 (14xCH_2_-alkyl), 20.9, 20.79, 20.77 (3xCH_3_-OAc), 18.9 (CH_3_-Um), 14.28 (CH_3_-alkyl). HRMS: calcd. for C_38_H_56_O_10_S [M+H]^+^ 705.3667, found: 705.3670.

**7-(((2*S*,3*R*,4*S*,5*S*,6*S*)-6-((Hexadecylthio)methyl)-3,4,5-trihydroxytetrahydro-2*H*-pyran-2-yl)oxy)-4-methyl-2*H*-chromen-2-one (9)**

The acetylated intermediate **25** (14 mg, 0.02 mmol) was dissolved in MeOH (3 mL) followed by the addition of sodium methoxide (30% in MeOH solution, 3 drops). The mixture was stirred for 4 h at room temperature after which TLC indicated complete conversion. The reaction was quenched with amberlite after which it was filtered and washed with MeOH. The solution was concentrated yielding the crude product which was purified by silica column chromatography (5% MeOH in CH_2_Cl_2_) yielding the desired product (19 mg, 0.03 mmol, 77% yield) as a white solid. ^1^H NMR (400 MHz, MeOD/CDCl_3_) *δ* 7.60 (d, *J* = 8.7 Hz, 1H, CH-Um), 7.11 (dd, *J* = 8.7, 2.4 Hz, 1H, CH-Um), 7.08 (d, *J* = 2.4 Hz, 1H, CH-Um), 6.17 (d, *J* = 1.3 Hz, 1H, CH-Um), 4.96 (d, *J* = 7.6 Hz, 1H, H-1), 3.56 (dd, *J* = 9.2, 7.8 Hz, 2H, H-2 and H-5), 3.47 (t, *J* = 9.0 Hz, 1H, H-4), 3.36 – 3.32 (m, 1H, H-3), 3.04 (dd, *J* = 14.4, 2.2 Hz, 1H, H-6a), 2.60 (dd, *J* = 14.3, 8.8 Hz, 1H, H-6b), 2.56 – 2.46 (m, 2H, CH_2_-alkyl), 2.43 (d, *J* = 1.2 Hz, 3H, CH_3_-Um), 1.48 (m, *J* = 7.5, 7.0 Hz, 2H, CH_2_-alkyl), 1.18 (m, *J* = 40.3 Hz, 26H, 13xCH_2_-alkyl), 0.89 – 0.81 (t, 3H, CH_3_-alkyl).; ^13^C NMR (101 MHz, MeOD/CDCl_3_) *δ* 162.9, 161.01, 155.3, 154.5 (4xCq-Um), 126.5 (CH-Um), 115.6 (Cq-Um), 114.5, 112.7, 104.8 (3xCH-Um), 101.1 (C-1), 78.6 (C-2), 77.0 (C-4), 74.0 (C-5), 73.5 (C-3), 34.0 (CH_2_-alkyl), 33.9 (C-6), 32.5, 30.23, 30.20, 30.15, 30.1, 29.9, 29.8, 29.5, 23.2 (14xCH_2_-alkyl), 18.9 (CH_3_-Um), 14.3 (CH_3_-alkyl). HRMS: calcd. for C_32_H_50_O_7_S [M+H]^+^ 579.3350, found: 579.3350.

**(2*R*,3*R*,4*S*,5*R*,6*S*)-2-(((*Tert*-butyldimethylsilyl)oxy)methyl)-6-(phenylthio)tetrahydro-2*H*-pyran-3,4,5-triyl triacetate (26)**

Intermediate **26** was synthesized over 5 steps starting from D-glucose following described procedures and its spectroscopic data was in agreement with the published literature (38, 39).

**(2*R*,3*R*,4*S*,5*R*,6*S*)-2-(Hydroxymethyl)-6-(phenylthio)tetrahydro-2*H*-pyran-3,4,5-triyl triacetate** **(27)**

Compound **26** (4.01 g, 7.82 mmol) was dissolved in a dry methanol and CH_2_Cl_2_ mixture (1:1 v/v 10 mL). To it a catalytic amount of *p*-TsOH (149 mg, 0.78 mmol) was added and the reaction was stirred for 2 h at room temperature. The reaction was quenched with triethylamine and washed with sat. NaHCO_3_ and brine. The combined water layers were washed an additional time with CH_2_Cl_2_ and the combined organic layers were dried over MgSO_4_, filtered and concentrated. The crude was purified by silica-gel column chromatography which yielded the product (2.79 g, 6.97 mmol, 89% yield) as a colorless syrup. ^1^H NMR (400 MHz, CDCl_3_) *δ* 7.46 (m, *J* = 6.5, 3.2 Hz, 2H, 2xCH_Ar_), 7.33 – 7.30 (m, 3H, 3xCH_Ar_), 5.25 (t, *J* = 9.4 Hz, 1H, H-3), 5.02 – 4.92 (m, 2H, H-2 and H-4), 4.73 (d, 1H, H-1), 3.73 (dd, *J* = 11.4, 6.8 Hz, 1H, H-6a), 3.62 – 3.52 (m, 2H, H-5 and H-6a), 2.07 (s, 3H, CH_3_-OAc), 2.03 (s, 3H, CH_3_-OAc), 1.99 (s, 3H, CH_3_-OAc). ^13^C NMR (101 MHz, CDCl_3_) *δ* 170.3, 170.1, 169.4 (3xCq-OAc), 133.0 (CH_Ar_), 131.7 (Cq-SPh), 129.2, 129.0 128.5, 128.3 (4xCH_Ar_), 85.7 (C-1), 78.7 (C-5), 73.9 (C-3), 70.2, 68.5 (C-2 and C-4), 61.6 (C-6). HRMS: calcd. for C_18_H_22_O_8_S [M+NH_4_]^+^ 416.1374, found: 416.1375.

**(2*R*,3*R*,4*S*,5*R*,6*S*)-2-((Hexadecylamino)methyl)-6-(phenylthio)tetrahydro-2*H*-pyran-3,4,5-triyl triacetate (28)**

Dess-martin periodinane (4.44 g, 10.5 mmol) was added to a solution of **27** (2.78 g, 6.97 mmol) in CH_2_Cl_2_ (25 mL). The mixture was stirred for 16 h at room temperature. The reaction mixture was quenched with sat. aq. Na_2_S_2_O_3_ and diluted with EtOAc. The organic layer was washed with sat. aq. NaHCO_3_ and H_2_O, dried over MgSO_4_, filtered, and concentrated *in vacuo*. The crude aldehyde was used in the next step without further purification. hexadecan-1-amine (2.53 g, 10.5 mmol) was dissolved in 2 mL anhydrous THF. The crude aldehyde (2.76 g, 6.97 mmol) was added to the solution and stirred for 2 h at room temperature. After 2 h the reaction mixture was cooled to 0 °C and a mixture of sodium cyanoborohydride (876 mg, 14.0 mmol) and AcOH (0.80 mL, 14.0 mmol) was added. After stirring overnight allowing to reach rt, the reaction was quenched with the addition of saturated aqueous ammonium chloride solution, and the mixture was extracted with ethyl acetate. The combined organic layers were washed with water, dried over MgSO_4_ and evaporated. The residue was purified by silica column chromatography yielding the product (735 mg, 1.18 mmol, 17% yield over 2 steps) as a yellow oil. ^1^H NMR (400 MHz, CDCl_3_) *δ* 7.46 (dd, *J* = 7.3, 2.3 Hz, 2H, CH-Ar), 7.35 – 7.28 (m, 3H, CH-Ar), 5.14 (t, *J* = 9.4 Hz, 1H, H-3), 5.06 (s, 1H, NH), 4.95 – 4.87 (m, 1H, H-2), 4.65 (d, *J* = 10.0 Hz, 1H, H-1), 4.26 – 4.16 (m, 1H, H-6a), 3.45 (dt, *J* = 9.8, 2.5 Hz, 1H, H-5), 3.41 – 3.24 (m, 3H, H-4 and CH_2_-alkyl), 3.18 (dd, *J* = 15.0, 2.5 Hz, 1H, H-6b), 2.15 (s, 3H, CH_3_-OAc), 2.08 (s, 3H, CH_3_-OAc), 2.05 (s, 3H, CH_3_-OAc), 1.59 (s, 2H, CH_2_-alkyl), 1.25 (s, 26H, CH_2_-alkyl), 0.92 – 0.84 (m, 3H, CH_3_-alkyl). ^13^C NMR (101 MHz, CDCl_3_) *δ* 173.3, 170.6, 169.9 (3xCq-OAc), 133.5 (CH-Ar), 131.9 (Cq-Ar), 129.1, 128.5 (2xCH-Ar), 86.2 (C-1), 80.7 (C-5), 74.9 (C-3), 70.6 (C-2), 68.3 (C-4), 51.1 (CH_2_-alkyl), 45.8 (C-6), 32.1, 29.8, 29.81, 29.77, 29.73, 29.70, 29.51, 29.45, 28.4, 27.0, 22.8 (14xCH_2_-alkyl), 21.2, 21.01, 20.99 (3xCH_3_-OAc), 14.3 (CH_3_-alkyl).; HRMS: calcd. for C_34_H_55_NO_7_S [M+H]^+^ 622.3772, found: 622.3774.

**7-(((2*S*,3*R*,4*S*,5*S*,6*R*)-6-((Hexadecylamino)methyl)-3,4,5-trihydroxytetrahydro-2*H*-pyran-2-yl)oxy)-4-methyl-2*H*-chromen-2-one (10)**

To a solution of the amino sugar **28** (735 mg, 1.18 mmol) and DIPEA (330 µL, 1.89 mmol) in THF (6.5 mL) benzyl chloroformate (253 µL, 1,77 mmol) was added dropwise at 0 °C. The temperature was raised to ambient after 10 minutes and stirring was continued until complete conversion of the starting material was observed after stirring the reaction overnight. All the volatiles were removed per rotatory evaporation. Excess reagent was flushed of over a small silica plug and the crude product was used in the next step without further purification. The crude Cbz protected compound (168 mg, 0.22 mmol) was dissolved in CH_2_Cl_2_ (2.8 mL), and cooled to 0 °C, in the dark, then bromine (17 µL, 0.33 mmol) was added. After 1.5 h the reaction mixture was diluted with CH_2_Cl_2_ and washed with 10% aqueous Na_2_S_2_O_3_, sat. aq. NaHCO_3_, and brine. The organic layer was dried over MgSO_4_, filtered, and concentrated. The crude bromide was used in the next step without further purification. 4MU (78 mg, 0.44 mmol) was added to a solution of NaOH (16.8 mg, 0.42 mmol) in water (2.2 mL). To this solution was added TBABr (72 mg, 0.22 mmol) and the crude intermediate sugar (161 mg, 0.22 mmol) dissolved in chloroform (2.2 mL). The mixture was stirred in the dark for 18 h at room temperature. After reaching completion the reaction was diluted with CH_2_Cl_2_ and washed with 1 M NaOH and brine. The water layers were extracted with CH_2_Cl_2_ and the combined organic layers were dried (MgSO_4_), filtered and concentrated under reduced pressure. The CBz protected amine crude was filtered over a short silica-gel plug leading to a mixture of product and some impurities which were deprotected in the next steps without further purification. This crude (37 mg) was deacetylated using the general procedure for acetyl deprotection with sodium methoxide. In a second step, Pd/C (100 mg, 0.09 mmol) was added to a stirred solution of the crude Cbz protected amino sugar (32 mg, 0.05 mmol) and AcOH (10 µL, 0.14 mmol) in ethanol/ethyl acetate (1/1, v/v, 4 mL). The reaction mixture was then purged with H_2_ and stirred for 1.5 h at rt. Upon completion, the solid was filtered off and the filtrate was concentrated *in vacuo*. The crude was purified using silica column chromatography (10% MeOH in CH_2_Cl_2_) yielding the product (11 mg, 0.02 mmol, 2% yield over 5 steps) as a white solid. ^1^H NMR (500 MHz, MeOD/CDCl_3_) *δ* 7.61 (d, *J* = 8.8 Hz, 1H, CH-Um), 7.08 (dd, *J* = 8.8, 2.5 Hz, 1H, CH-Um), 7.00 (d, *J* = 2.4 Hz, 1H, CH-Um), 6.17 (d, *J* = 1.2 Hz, 1H, CH-Um), 5.08 (d, *J* = 7.4 Hz, 1H, H-1), 3.80 – 3.72 (m, 1H, H-5), 3.56 – 3.48 (m, 2H, H-2 and H-3), 3.30 – 3.25 (m, 2H, H-4 and H-6a), 2.95 (dd, *J* = 13.1, 8.4 Hz, 1H, H-6b), 2.83 – 2.73 (m, 2H, CH_2_-alkyl), 2.43 (s, 3H, CH_3_-Um), 1.60 – 1.49 (m, 2H, CH_2_-alkyl), 1.20 (d, *J* = 22.0 Hz, 26H, 13xCH_2_-alkyl), 0.88 – 0.82 (t, 3H, CH_3_-alkyl). ^13^C NMR (126 MHz, MeOD/CDCl_3_) *δ* 162.6, 160.6, 155.6, 154.2 (4xCq-Um), 126.6 (CH-Um), 115.5 (Cq-Um), 114.1, 112.7, 104.3 (3xCH-Um), 100.4 (C-1), 76.5 (C-2), 73.6 (C-3), 73.5 (C-5), 72.4 (C-4), 49.8 (C-6), 49.6, 32.4, 30.10, 30.07, 30.05, 30.0, 29.9, 29.8, 29.7, 27.2, 23.1 (15xCH_2_-alkyl), 18.9 (CH_3_-Um), 14.3 (CH_3_-alkyl). HRMS: calcd. for C_32_H_51_NO_7_ [M+H]^+^ 562.3738, found: 562.3739.

**(2*R*,3*R*,4*S*,5*R*,6*R*)-2-((Benzoyloxy)methyl-^13^C)-6-(2,2,2-trichloro-1-iminoethoxy)tetrahydro-2*H*-pyran-3,4,5-triyl-2,3,4,5,6-^13^C5 tribenzoate (29)**

To a solution of ^13^C_6_-D-glucose (1.0 g, 5.55 mmol) in pyridine (100 mL) at 0 °C, BzCl (3.87 mL, 33.3 mmol) and a catalytic amount of DMAP (68 mg, 0.56 mmol) were added. The reaction was stirred for 20 h at room temperature after which the mixture of the reaction was diluted with EtOAc (100 mL) and washed with water (1 x 100 mL) and a 1M HCl (3 x 100 mL). The organic layer was dried over MgSO_4_, filtered and concentrated *in vacuo*. The crude product was recrystallized from ethanol and immediately used in the next step. The benzoylated intermediate (2.5 g, 3.57 mmol) was dissolved in dry THF (7.14 mL) and was cooled to 0 °C under argon atmosphere. DMAPA (7.25 mL, 10.7 mmol) is added to the reaction flask and is stirred for 2.5 hours at 0 °C. Upon completion of the reaction the mixture was diluted with 75 mL EtOAc and the organic layer was washed twice with 75 mL 1 M HCL and brine. The combined water layers were extracted with EtOAc and the combined organic layers were dried with MgSO_4_, filtered and then concentrated *in vacuo*. The crude intermediate (2.13 g, 3.57 mmol) is dissolved in dry CH_2_Cl_2_ (12 mL) under argon atmosphere and CCl_3_CN (1.07 mL, 10.7 mmol) is added to the reaction flask. Cs_2_CO_3_ powder (465 mg, 1.43 mmol) is then added to the solution and the reaction mixture is left to stir for 3.5 hours at rt. The solution was then diluted with 75 mL of CH_2_Cl_2_ and washed twice with 100 mL NaHCO_3_ and once with brine. The organic layers are dried with MgSO_4_, filtered and then concentrated *in vacuo*. The crude mixture is purified on a silica column (15:5 pentane: EtOAc + 2% triethylamine). Compound **29** (2.03 g, 2.73 mmol, 77% yield over 3 steps) is collected as a yellowish oil. ^1^H NMR (400 MHz, CDCl_3_) *δ* 8.66 (s, 1H, NH), 8.08 – 8.03 (m, 2H, CH-OBz), 8.00 – 7.94 (m, 4H, CH-OBz), 7.91 – 7.86 (m, 2H, CH-OBz), 7.56 (t, *J* = 7.4 Hz, 1H, CH-OBz), 7.51 (t, *J* = 7.5 Hz, 2H, CH-OBz), 7.43 (t, *J* = 7.9 Hz, 3H, CH-OBz), 7.36 (d, *J* = 7.7 Hz, 4H, CH-OBz), 7.30 (t, *J* = 7.8 Hz, 2H, CH-OBz), 6.86 (d, *J* = 3.5 Hz, 1H, H-1), 6.30 (t, *J* = 10.2 Hz, 1H, H-3), 5.85 (t, *J* = 10.2 Hz, 1H, H-4), 5.65 (dd, *J* = 10.1, 3.5 Hz, 1H, H-2), 4.66 (d, *J* = 11.0 Hz, 2H, H-5 and H-6a), 4.51 (dd, *J* = 12.5, 4.8 Hz, 1H, H-6b). ^13^C NMR (101 MHz, CDCl_3_) *δ* 166.1, 165.7, 165.5, 165.3 (4xCq-OBz), 160.6 (Cq-Imidate), 1337, 133.4, 133.3, 130.0, 129.9, 129.8 (6xCH-OBz), 129.6, 128.9, 128.7, 128.6 (4xCq-OBz), 128.54, 128.48, 128.45 (CH-OBz), 93.2 (d, *J* = 44.7 Hz, ^13^C-1), 71.4 – 69.7 (m, ^13^C-2, ^13^C-3 and ^13^C-5), 69.2 – 68.1 (m, ^13^C-4), 62.5 (d, *J* = 44.2 Hz, ^13^C-6). HRMS: calcd. for C_30_^13^C_6_H_28_Cl_3_NO_10_ [M+H]^+^ 768,08723, found: 768,08723.

**(2*R*,3*R*,4*S*,5*R*,6*R*)-2-((Benzoyloxy)methyl-^13^C)-6-(((3*R*,8*S*,9*S*,10*R*,13*R*,14*S*,17*R*)-10,13-dimethyl-17-((*R*)-6-methylheptan-2-yl)-2,3,4,7,8,9,10,11,12,13,14,15,16,17-tetradecahydro-1*H*-cyclopenta[*a*]phenanthren-3-yl)oxy)tetrahydro-2*H*-pyran-3,4,5-triyl-2,3,4,5,6-^13^C5 tribenzoate (30)**

The imidate sugar donor **29** (150 mg, 202.4 μmol) was dissolved in dry CH_2_Cl_2_ (2 mL) and cholesterol (94 mg, 242.9 μmol) was added at 0 °C. The reaction mixture was stirred at 0 °C on activated 3 Å molecular sieves under argon and boron trifluoride etherate (8 μL, 60 μmol) was added and stirring was maintained for 3 h. After neutralization with triethylamine (10 µL) and filtration over celite, the mixture was evaporated under reduced pressure and purified by silica gel chromatography giving compound **30** (51 mg, 71 μmol, 35%) as an off-white solid. ^1^H NMR (400 MHz, CDCl_3_) *δ* 8.04 – 7.99 (m, 2H, CH-OBz), 7.98 – 7.93 (m, 2H, CH-OBz), 7.93 – 7.88 (m, 2H, CH-OBz), 7.86 – 7.80 (m, 2H, CH-OBz), 7.57 – 7.47 (m, 3H, CH-OBz), 7.45 – 7.25 (m, 9H, CH-OBz), 5.90 (t, *J* = 9.5 Hz, 1H, CH-Chol), 5.63 (t, *J* = 9.5 Hz, 1H, H-3), 5.55 – 5.45 (t, 1H, H-4), 5.23 (d, *J* = 5.6 Hz, 1H, H-2), 4.94 (d, *J* = 7.7 Hz, 1H, H-1), 4.60 (dd, *J* = 11.8 Hz, 1H, H-6a), 4.52 (dd, *J* = 10.7, 4.5 Hz, 1H, H-6b), 4.20 – 4.10 (m, 1H, H-5), 3.53 (td, *J* = 10.6, 5.8 Hz, 1H, CH-Chol), 2.17 (m, *J* = 5.4 Hz, 2H, CH_2_-Chol), 2.06 – 1.97 (m, 1H, CH-Chol), 1.96 – 1.88 (m, 2H, CH_2_-Chol), 1.86 – 1.77 (m, 1H, CH-Chol), 1.71 (m, 1H), 1.58 – 1.47 (m, 3H, CH_2_-Chol and CH-Chol), 1.45 – 1.30 (m, 8H, CH_2_-Chol and CH-Chol), 1.26 (s, 3H, CH_3_-Chol), 1.19 – 0.95 (m, 9H, CH_2_-Chol and CH-Chol), 0.92 (m, 2H, CH-Chol), 0.90 (s, 3H, CH_3_-Chol), 0.87 (d, *J* = 1.8 Hz, 3H, CH_3_-Chol), 0.86 (d, *J* = 1.8 Hz, 3H, CH_3_-Chol), 0.65 (s, 3H, CH_3_-Chol). ^13^C NMR (101 MHz, CDCl_3_) *δ* 166.2, 166.0, 165.4, 165.2 (4xCq-OBz), 140.5 (Cq-Chol), 133.6, 133.34, 133.29, 133.2 (4xCH-OBz), 130.0, 129.91, 129.88, 129.85 (4xCH-OBz), 129.8, 129.6, 129.0, 128.9 (4xCq-OBz), 128.54, 128.50, 128.47, 128.4 (4xCH-OBz), 122.1 (CH-Chol), 100.3 (d, *J* = 49.3 Hz, C-1), 95.2, 94.7 (2xCq-Chol), 80.6 (CH-Chol), 74.0 – 71.5 (m, ^13^C-2, ^13^C-3 and ^13^C-5)), 70.8 – 69.5 (m, ^13^C-4), 63.5 (d, *J* = 44.6 Hz, ^13^C-6), 56.9, 56.3, 50.2 (3xCH-Chol), 42.4, 39.9, 39.6, 38.9, 37.2, 36.7, 36.3 (7xCq and CH_2_-Chol), 35.9 (CH-Chol), 32.0 (CH_2_-Chol), 31.9 (CH-Chol), 29.8, 29.7, 28.4 (3xCq or CH_2_-Chol), 28.2 (3xCH or CH_3_-Chol), 24.4, 23.9 (2xCq or CH_2_-Chol), 23.0, 22.7 (2xCH_3_-Chol), 21.1 (CH_2_-Chol), 19.4, 18.9, 12.0 (3xCH_3_-Chol). HRMS: not detected. LCMS: calcd. for C_55_^13^C_6_H_72_O_10_ [M+NH_4_]^+^ 988.57, found: 988.5

**((2*R*,3*S*,4*S*,5*R*,6*R*)-6-(((3*R*,8*S*,9*S*,10*R*,13*R*,14*S*,17*R*)-10,13-Dimethyl-17-((*R*)-6-methylheptan-2-yl)-2,3,4,7,8,9,10,11,12,13,14,15,16,17-tetradecahydro-1*H*-cyclopenta[*a*]phenanthren-3-yl)oxy)-3,4,5-trihydroxytetrahydro-2*H*-pyran-2-yl-2,3,4,5,6-^13^C5)methyl-^13^C-palmitate (31)**

Intermediate **30** (205 mg, 212 μmol) was dissolved in a mixture of MeOH (2.1 mL) and CH_2_Cl_2_ (2.1 mL) followed by the addition of a catalytic amount of sodium methoxide (30% in MeOH, 1 drop). The mixture was stirred for 4 h at room temperature. After reaching completion the reaction was quenched with amberlite, which was subsequently filtered off and washed with MeOH and CH_2_Cl_2_. The solution was concentrated *in vacuo* yielding the crude intermediate which was used in the next step without further purification. Palmitic acid (47 mg, 181 μmol), HATU (69 mg, 181 μmol), and diisopropyl ethylamine (53 μL, 302 μmol) were dissolved in dry pyridine (2 mL) and the resulting mixture was stirred at rt for 30 minutes under a nitrogen atmosphere. The crude deprotected ^13^C_6_-Glucosylated cholesterol intermediate (83 mg, 151 μmol) was dissolved in pyridine (1 mL) was then injected into the reaction mixture via syringe and stirring was continued at rt for 72 h. The pyridine was removed *in vacuo* and the resulting solid was purified and residual starting material recovered using silica column chromatography to give a white solid (32 mg, 88% yield brsm). ^1^H NMR (400 MHz, MeOD/CDCl_3_) *δ* 5.33 (d, *J* = 5.4 Hz, 1H, CH-Chol), 4.39 – 4.31 (m, 2H, H-1 and H-6a), 4.19 (dd, *J* = 11.7, 7.0 Hz, 1H, H-6b), 3.51 (m, 1H, CH-Chol), 3.44 (t, *J* = 8.3 Hz, 1H, H-5), 3.36 (t, *J* = 8.9 Hz, 1H, H-3), 3.23 (dt, *J* = 16.6, 8.9 Hz, 2H, H-2 and H-4), 2.36 (m, 1H, CH-Chol), 2.29 (t, *J* = 7.7 Hz, 2H), 2.27 – 2.21 (m, 1H, CH-Chol), 2.03 – 1.87 (m, 3H, CH-Chol and CH_2_-Chol), 1.81 (m, 2H, CH_2_-Chol), 1.65 – 1.53 (m, 4H, CH_2_-pamityl and CH_2_-Chol), 1.52 – 1.40 (m, 4H, CH_2_-Chol), 1.23 (m, 30H, 13xCH_2_-palmityl and 2xCH_2_-Chol), 1.15 – 1.02 (m, 6H, CH-Chol and CH_2_-Chol), 0.98 (m, 5H, CH_3_-Chol and CH_2_-Chol), 0.91 – 0.86 (m, 4H, CH-Chol and CH_3_-Chol), 0.85 – 0.83 (m, 4H, CH-Chol and CH_3_-Chol), 0.82 (d, *J* = 1.7 Hz, 3H, CH_3_-Chol), 0.65 (s, 3H, CH_3_-Chol). ^13^C NMR (101 MHz, MeOD/CDCl_3_) *δ* 174.9 (Cq-Palmityl), 141.0 (Cq-Chol), 122.4 (CH-Chol), 101.8 (dt, *J* = 47.1, 4.2 Hz, ^13^C-1), 97.6, 97.1 (2xCq-Chol), 80.1 (CH-Chol), 77.0 (t, *J* = 39.2 Hz, ^13^C-3), 74.8 – 73.1 (m, ^13^C-5 and ^13^C-2 or ^13^C-4), 70.2 (t, *J* = 40.0 Hz, ^13^C-2 or ^13^C-4), 64.3 (dt, *J* = 45.3, 4.5 Hz, ^13^C-6), 57.2, 56.6, 50.6 (3xCH-Chol), 42.7, 40.2, 39.9, 39.1, 37.8, 37.1, 36.6 (7xCH_2_-Chol), 36.2 (CH-Chol), 34.7 (CH_2_-Chol), 32.3 (CH_2_-Chol), 32.3 (CH-Chol), 30.13, 30.10, 30.06, 30.0, 29.78, 29.75, 29.7, 28.6 (8xCH_2_-Chol), 28.4 (CH-Chol), 25.4, 24.6, 24.2, 23.0 (4xCH_2_-Chol), 23.0 (CH_3_-Chol), 22.7 (CH-Chol), 21.5 (CH_2_-Chol), 20.0, 19.0, 14.3, 12.1 (4xCH_3_-Chol). HRMS: not detected. LCMS: calcd. for C_55_^13^C_6_H_72_O_10_ [M+Na]^+^ 815.65, found: 815.6

4. NMR Spectra **(2) measured in CDCl_3_**

**(3) measured in CDCl_3_**

**(4) measured in CDCl_3_**

**(5) measured in CDCl_3_**

**(12) measured in CDCl_3_**

**(13) measured in CDCl_3_**

**(14) measured in CDCl_3_**

**(9) measured in MeOD/CDCl_3_**

**(15) measured in CDCl_3_**

**(16) measured in CDCl_3_**

**(17) measured in CDCl_3_**

**(18) measured in CDCl_3_**

**(19) measured in CDCl_3_**

**(20) measured in CDCl_3_**

**(21) measured in CDCl_3_**

**(6) measured in MeOD/CDCl_3_**

**(22) measured in CDCl_3_**

**(23) measured in CDCl_3_**

**(24) measured in MeOD**

**(7) measured in MeOD/CDCl_3_**

**(25) measured in CDCl_3_**

**(8) measured in MeOD/CDCl_3_**

**(27) measured in CDCl_3_**

**(28) measured in CDCl_3_**

**(10) measured in MeOD/CDCl_3_**

**(29) measured in CDCl_3_**

**(30) measured in CDCl_3_**

**(31) measured in MeOD/CDCl_3_**

5. References

24. Saehlim, N., Athipornchai, A., Sirion, U., & Saeeng, R. (2020). New class of alkynyl glycoside analogues as tyrosinase inhibitors. *Bioorganic & medicinal chemistry letters*, *30*(15), 127276. https://doi.org/10.1016/j.bmcl.2020.127276

36. Lelieveld LT, Mirzaian M, Kuo CL, Artola M, Ferraz MJ, Peter REA, Akiyama H, Greimel P, van den Berg RJBHN, Overkleeft HS, Boot RG, Meijer AH, Aerts JMFG. Role of β-glucosidase 2 in aberrant glycosphingolipid metabolism: model of glucocerebrosidase deficiency in zebrafish. J Lipid Res. 2019 Nov;60(11):1851-1867. doi: 10.1194/jlr.RA119000154. Epub 2019 Sep 27. PMID: 31562193; PMCID: PMC6824494.

37. Gu, Z., Eils, R., & Schlesner, M. (2016). Complex heatmaps reveal patterns and correlations in multidimensional genomic data. *Bioinformatics (Oxford, England)*, *32*(18), 2847–2849. https://doi.org/10.1093/bioinformatics/btw313.

38. Dussouy, C., Téletchéa, S., Lambert, A., Charlier, C., Botez, I., De Ceuninck, F., & Grandjean, C. (2020). Access to Galectin-3 Inhibitors from Chemoenzymatic Synthons. *The Journal of organic chemistry*, *85*(24), 16099–16114. https://doi.org/10.1021/acs.joc.0c01927

39. Michihata, N., Kaneko, Y., Kasai, Y., Tanigawa, K., Hirokane, T., Higasa, S., & Yamada, H. (2013). High-yield total synthesis of (-)-strictinin through intramolecular coupling of gallates. *The Journal of organic chemistry*, *78*(9), 4319–4328. https://doi.org/10.1021/jo4003135
